# Annual Hospitalizations for COVID-19, Influenza, and Respiratory Syncytial Virus, United States, 2023–2024

**DOI:** 10.3201/eid3103.240594

**Published:** 2025-03

**Authors:** Kaiming Bi, Shraddha Ramdas Bandekar, Anass Bouchnita, Spencer J. Fox, Lauren Ancel Meyers

**Affiliations:** The University of Texas at Austin, Austin, Texas, USA (K. Bi, S.R. Bandekar, L. Ancel Meyers); The University of Texas at El Paso, El Paso, Texas, USA (A. Bouchnita); University of Georgia, Athens, Georgia, USA (S.J. Fox)

**Keywords:** COVID-19, SARS-CoV-2, influenza, respiratory syncytial virus, modeling, scenario projection, viruses, respiratory infections, zoonoses, vaccine-preventable diseases, United States

## Abstract

Projections for the US 2023–24 respiratory virus season indicated a 31% decrease to a 55% increase in hospitalizations for respiratory syncytial virus, influenza, and COVID-19 compared with 2022–23, depending on circulating variants and vaccination uptake. The projections captured the tripledemic peak but missed the multiwave seasonality of COVID-19.

Nonpharmaceutical interventions to combat the COVID-19 pandemic disrupted the seasonal transmission of influenza virus and respiratory syncytial virus (RSV). During November 2022–March 2023, co-circulation of SARS-CoV-2, influenza virus, and RSV caused a tripledemic that strained US healthcare systems. By the summer of 2023, global authorities had officially declared the end of the COVID-19 pandemic (*1*), and the recent tripledemic had bolstered populationwide immunity against the 3 viruses, leading to considerable uncertainty about the potential severity of the 2023–24 respiratory virus season and the effects of updated vaccines for SARS-CoV-2 and influenza (*2*), the novel Food and Drug Administration–approved RSV vaccine for older adults and pregnant women, and the novel monoclonal antibody therapy for infants with RSV (*3*). To support national planning, we created a series of scenario projections by using validated models of influenza, SARS-CoV-2, and RSV that track immunity stemming from infection and immunization (Appendix) and compared projected 2023–24 hospitalizations to those reported during the 2022–23 season. We retrospectively evaluated the validity of the assumptions and the consistency of the projections compared with actual epidemiologic trends in the United States during September 1, 2023–March 30, 2024.

The 4 SARS-CoV-2 scenarios assumed that an immune-evasive EG.5-like variant either would or would not emerge in May 2023 and that updated booster vaccines would be recommended for everyone or only for adults >65 years of age (Appendix). In retrospect, the scenarios optimistically assumed 52% booster coverage for persons >65 years of age and 34% for persons <65 years of age, which are almost double the reported uptake of 28.1% for persons >65 years of age and 19.1% for persons <65 years of age (*4*). Moreover, none of the scenarios anticipated the October emergence of the highly transmissible JN.1 variant (*5*). Our projections of 598,000 (95% CI 486,000–692,000) to 1,310,000 (95% CI 1,089,000–1,493,000) total COVID-19 hospitalizations were high compared with the 612,616 eventually reported, and the projected mid-October peak was considerably earlier than the actual December peak (Figure, panel A). Our model cannot explain the persistent 2-wave seasonal dynamics of COVID-19, which could stem from frequent emergence of new variants, durability of infection-acquired and vaccine-acquired immunity, or other unidentified seasonal factors.

**Figure:**
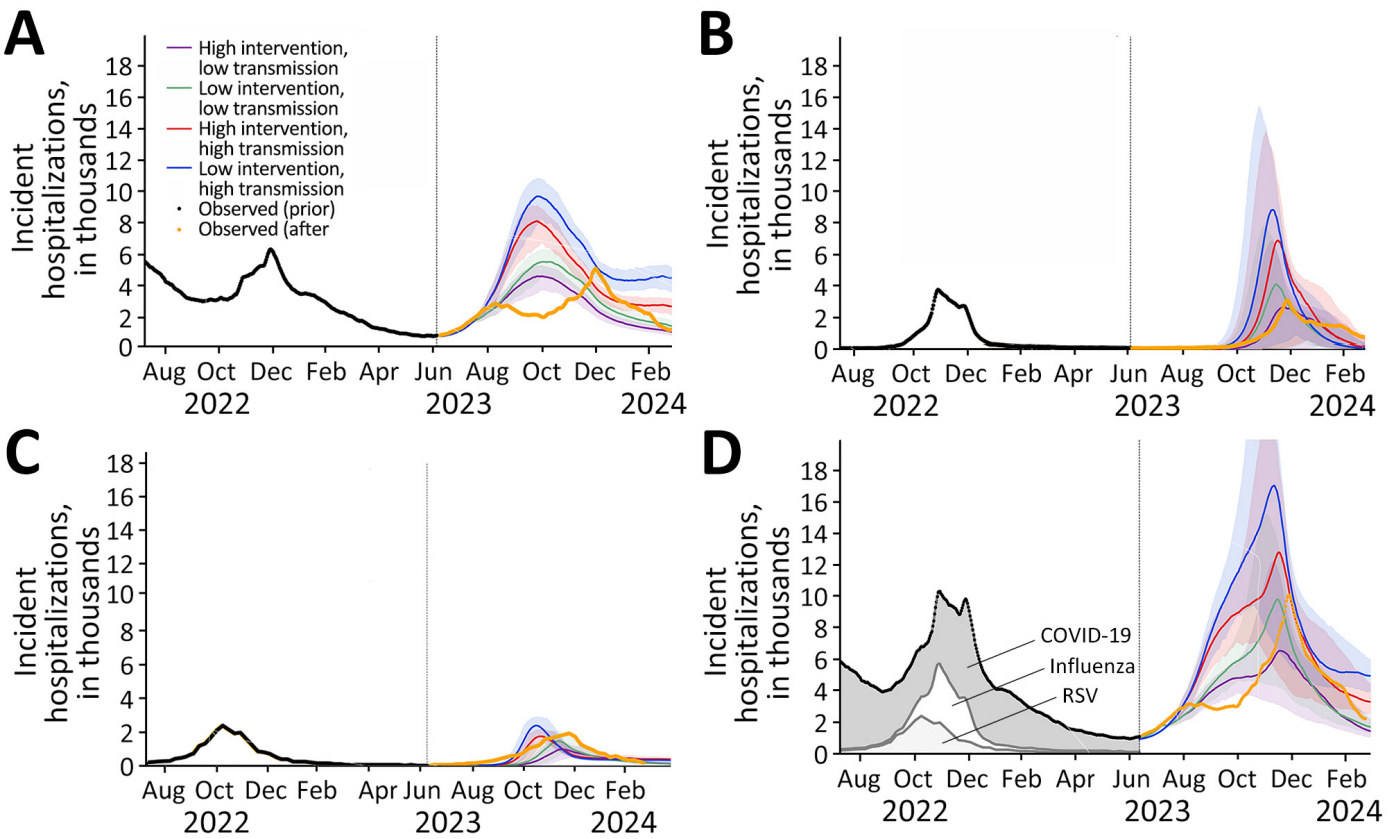
Projected daily hospital admissions attributable to COVID-19 (A), influenza (B), RSV (C), and COVID-19, influenza, or RSV infections combined (D) under multiple scenarios with varying viral transmission rates and varying effect of medical countermeasures, United States, June 8, 2023–March 30, 2024. Values are the 7-day average number of hospital admissions attributable to infections by the specified viruses. The solid lines indicate medians; shaded ribbons indicate 95% CIs across 200 stochastic simulations. Black dots in all graphs and gray shading in panel D indicate the reported 7-day average hospital admissions for the 3 viruses before the projection period (*9*,*10*); orange lines indicate 7-day average hospital admissions from the projection period that were not available at the time of the initial analysis. RSV, respiratory syncytial virus.

The influenza scenarios vary in terms of the dominant subtype (H1N1 vs. H3N2) and national vaccination coverage (40% vs. 60%) (Appendix). In retrospect, the 2023–24 influenza season was dominated by influenza A(H1N1), and an estimated 47.3% of the US population received an influenza vaccine (*6*). Under the best-matching scenario (H1N1 with 40% coverage), daily influenza hospital admissions were projected to peak on December 16 (95% CI November 21–January 25) at 4,100 (95% CI 400–8,700) and to total 210,000 (95% CI 17,000–475,000) (Figure, panel B). In reality, influenza hospital admissions peaked on December 28 at 3,137 and totaled 215,667. Comparing our 2 influenza A(H1N1) scenarios, we estimated that vaccinating 60% versus 40% of the US population would have prevented 27,000 (95% CI 6,000–34,000) influenza-related hospitalizations.

The RSV scenarios assume either a high transmission rate (estimated in 2022–23) or a low transmission rate (estimated before 2020) and either high or low vaccination uptake. Under the high uptake scenario, the new RSV vaccine is recommended for persons >60 years of age (assuming 56.12% uptake based on 2022–23 influenza vaccination coverage), and all infants <8 months of age receive the new monoclonal antibody (Appendix). As of April 2024, only ≈23.6% (95% CI 22.7%–24.5%) of older adults had received the vaccine and 43.0% (95% CI 33.9%–52.1%) of infants had received antibody injections (*7*,*8*). By fitting our model to 2023–24 RSV hospitalization data (Appendix), we estimated that the transmission rate for children <18 years of age was similar to prepandemic levels, whereas that for older persons might have been higher than during the 2022–23 season. Although our scenarios did not anticipate this complexity, the observed trends are roughly consistent with the projections. The scenario assuming a prepandemic transmission rate and low vaccination uptake projected a total of 95,000 (95% CI 52,000–157,000) RSV hospitalizations, peaking on December 12 (95% CI December 5–25) with 1,500 (95% CI 800–2,100) daily admissions. In reality, US RSV hospital admissions peaked on December 30 at 1,911 and totaled 178,000 (Figure, panel C).

We aggregated the individual pathogen scenario projections to estimate overall tripledemic hospitalizations, assuming no epidemiologic interactions among them (Appendix). The subsequently observed trends fell well within the projected CIs (Figure, panel D) and were closest to the scenario assuming low transmission rates and low vaccination uptake for all 3 viruses. Although the scenario assumptions were imperfect, particularly for COVID-19, the projected cumulative hospitalizations of 1,029,000 (95% CI 688,000–1,518,000) and early December peak of 9,800 (95% CI 4,700–15,246) daily admissions are consistent with the observed 1,007,000 total hospitalizations and late December peak of 10,082 (Appendix). Even when the assumptions prove wrong, simulating a range of carefully constructed scenarios can help anticipate the timing and severity of epidemics, assess the probable effect of interventions, and guide healthcare capacity planning.

Appendix Additional information about annual hospitalizations for COVID-19, influenza, and respiratory syncytial virus, United States, 2023–2024.

## References

[1] World Health Organization. Statement on the fifteenth meeting of the IHR (2005) Emergency Committee on the COVID-19 pandemic. 2023 May 5 [cited 2023 Aug 13]. https://www.who.int/news/item/05-05-2023-statement-on-the-fifteenth-meeting-of-the-international-health-regulations-(2005)-emergency-committee-regarding-the-coronavirus-disease-(covid-19)-pandemic

[2] Kumari M, Lu RM, Li MC, Huang JL, Hsu FF, Ko SH, et al. A critical overview of current progress for COVID-19: development of vaccines, antiviral drugs, and therapeutic antibodies. J Biomed Sci. 2022;29:68. 10.1186/s12929-022-00852-9 36096815 PMC9465653

[3] Food and Drug Administration. Respiratory syncytial virus (RSV). 2024 Oct 22 [cited 2024 Sep 4]. https://www.fda.gov/consumers/covid-19-flu-and-rsv/respiratory-syncytial-virus-rsv

[4] Centers for Disease Control and Prevention. Weekly COVID-19 vaccination dashboard. 2025 Jan 15 [cited 2024 Sep 4]. https://www.cdc.gov/vaccines/imz-managers/coverage/covidvaxview/interactive/vaccination-dashboard.html

[5] Kaku Y, Okumura K, Padilla-Blanco M, Kosugi Y, Uriu K, Hinay AA Jr, et al.; Genotype to Phenotype Japan (G2P-Japan) Consortium. Virological characteristics of the SARS-CoV-2 JN.1 variant. Lancet Infect Dis. 2024;24:e82. 10.1016/S1473-3099(23)00813-7 38184005

[6] Centers for Disease Control and Prevention. Influenza vaccine doses distributed. 2025 Jan 15 [cited 2023 Sep 4]. https://www.cdc.gov/flu/fluvaxview/dashboard/vaccination-doses-distributed.html

[7] Centers for Disease Control and Prevention. Infant protection against respiratory syncytial virus (RSV) by maternal RSV vaccination or receipt of nirsevimab, and intent for nirsevimab receipt, United States. 2025 Jan 15 [cited 2023 Aug 13]. https://www.cdc.gov/vaccines/imz-managers/coverage/rsvvaxview/nirsevimab-coverage.html.

[8] Centers for Disease Control and Prevention. Respiratory syncytial virus (RSV) vaccination coverage and intent for vaccination, adults 75 years and older and adults 60–74 years with high-risk conditions, United States. 2025 Jan 15 [cited 2024 Sep 4]. https://www.cdc.gov/vaccines/imz-managers/coverage/rsvvaxview/adults-60-coverage-intent.html

[9] US Department of Health and Human Services. COVID-19 reported patient impact and hospital capacity by state timeseries (raw). 2024 Jun 28 [cited 2024 May 3]. https://healthdata.gov/Hospital/COVID-19-Reported-Patient-Impact-and-Hospital-Capa/g62h-syeh/about_data

[10] Centers for Disease Control and Prevention. RSV-NET interactive dashboard 2024 Oct 10 [cited 2024 Sep 4]. https://www.cdc.gov/rsv/research/rsv-net/dashboard.html

