# Correction: Vol. 31, No. 3

**DOI:** 10.3201/eid3105.C13105

**Published:** 2025-05

**Keywords:** errata, erratum, correction

The author list was incorrect in Larone’s Medically Important Fungi: A Guide to Identification, 7th Edition (M.M. Azar). The article has been corrected online (https://wwwnc.cdc.gov/eid/article/31/3/23-1623_article).

