# Correction: Vol. 31, No. 3

**DOI:** 10.3201/eid3105.C23105

**Published:** 2025-05

**Keywords:** errata, erratum, correction

A grant was missing from the funding list in Annual Hospitalizations for COVID-19, Influenza, and Respiratory Syncytial Virus, United States, 2023–2024 (K. Bi et al.). The article has been corrected online (https://wwwnc.cdc.gov/eid/article/31/3/24-0594_article).

