# Supplementary material for: Annual Hospitalizations for COVID-19, Influenza, and Respiratory Syncytial Virus, United States, 2023–2024
Source: Emerg Infect Dis. 2025 Mar;31(3):636–8. doi: 10.3201/eid3103.240594 (PMC11878295; doi:10.3201/eid3103.240594)
Supplement: Appendix — Additional information about annual hospitalizations for COVID-19, influenza, and respiratory syncytial virus, United States, 2023–2024. [file 24-0594-Techapp-s1.pdf]

Article DOI: https://doi.org/10.3201/eid3103.240594

EID cannot ensure accessibility for supplementary materials supplied by authors. Readers who have difficulty accessing supplementary content should contact the authors for assistance.

Annual Hospitalizations for COVID-19, Influenza, and Respiratory Syncytial Virus, United States, 2023–2024

Appendix

Supplementary Material A: 2023-2024 Respiratory Virus Season Scenarios

A.1 SARS-CoV-2 scenarios for projecting infection-related hospitalizations during the 2023-2024 respiratory virus season

The four SARS-CoV-2 scenarios for the 2023-2024 respiratory virus season (**Appendix Table 1**) each assume one of two possibilities for SARS-CoV-2 variants: (i) no new variant emerges during the projection period and susceptibility is driven solely by immune waning and seasonal forcing, or (ii) the EG.5 variant emerges at a rate estimated from genomic surveillance data between May 13 and August 19, 2023[1] (**Appendix Figure 1**). We assume that immunity previously acquired from vaccination and infection by previously circulating variants is 50% less effective against the EG.5 variant than the prior XBB 1.5 variant[2]. Each scenario also considers one of two possibilities for vaccine policy: (i) the new SARS-CoV-2 booster that includes the XBB.1.5 COVID-19 variant is recommended for all age groups, or (ii) the vaccine is recommended for only those over the age of 65. In both cases, we assume that the vaccine efficacy is 65% against both symptomatic and severe disease[3], the booster first becomes available on October 1, 2023, and that age-specific rates of vaccination for the recommended groups will be comparable to uptake during the first SARS-CoV-2 booster vaccine campaign in September 2021 (e.g., reaching 19.50% of individuals over 65 in the first two months)[4].

A.2 Influenza scenarios for projecting infection-related hospitalizations during the 2023-2024 respiratory virus season

The four influenza scenarios for the 2023-2024 respiratory virus season (**Appendix Table 2**) each assume one of two possibilities for the dominant influenza subtype: (i) H3N2 (which dominated during the 2022-2023 influenza season[5]) or (ii) H1N1 (which dominated during the recent 2023 influenza season in the Southern Hemisphere[6]). For the H1N1-dominant scenario, we estimated the transmissibility and severity based on data from the 2019-2020 season in the US[7], for the H3N2-dominant scenario, we based our estimates on data from 2017-2018 season, which was

anomalously severe[8]. Each scenario also considers one of two possibilities for age-specific vaccine coverage: (i) lower uptake, as in 2011-2012 season (reaching ~40% coverage) or (ii) higher uptake, as in 2020-2021 season, (reaching ~60% coverage)[9]. For comparison, an estimated 51% of the US population received an influenza vaccine during the 2022-2023 season[9]. In both cases, we assume that vaccination reduces the risk of influenza hospitalization by 40%, based on the average vaccine efficacy estimated across the 2011-2023 influenza seasons[10]. Age-specific vaccination rates are drawn from 2021-2022 influenza season, vaccine coverage is 20% higher than that of the 2021-2022 season for the high vaccine uptake scenario, vaccine coverage is 20% lower than that of the 2021-2022 season for the low vaccine uptake scenario[11].

A.3 RSV scenarios for projecting infection-related hospitalizations during the 2023-2024 respiratory virus season

The four RSV scenarios for the 2023-2024 respiratory virus season (**Appendix Table 3**) each assume one of two possibilities for the transmissibility of RSV: (i) transmission rates return to lower pre-pandemic values estimated based on data from the 2018-2019 and 2019-2020 RSV seasons in the US[12] or (ii) they remain elevated at values estimated based on data from the 2022-2023 season in the US[12] (which are consistent with RSV transmission rates during more recent 2023 season in the Southern Hemisphere[6]). Each scenario also assumes one of two possibilities for the impact of recently FDA-approved vaccines and monoclonal antibodies to protect against RSV: (i) the Arexy RSV vaccine[13] is *not recommended* for any age group and the Beyfortus monoclonal antibody[14] is recommended *only for high-risk infants* under eight months or (ii) Arexvy is recommended for *all individuals over age 60* and the Beyfortus immunization is recommended for *all infants* under eight months. In the latter case, we assume Arexvy has an efficacy of 94.1% against RSV hospitalization[15]. The efficacy of Arexvy against infection remains uncertain, with some estimates suggesting no efficacy[16] and others indicating a high level of efficacy[17]. As such, we assume that protection against infection follows a uniform distribution ranging from 0% to 71.7%[15]. We further assume that vaccination begins on September 1, 2023, based on an early estimate for the timing of the campaign. The vaccination rate among people over 60 is estimated to be comparable to the uptake of the influenza vaccine during the 2022-2023 season. We assume that Beyfortus reduces the chances of RSV-related hospitalization by 62.1% and the chance of RSV infection in infants similarly follows a uniform distribution ranging from0% to 74.5%[18], that it also becomes available on September 1, 2023, with almost 50% of the target population receiving an injection by November 1, 2023[18].

A.4 Combined influenza, SARS-CoV-2 and RSV scenarios for projecting infection-related hospitalizations during the 2023-2024 respiratory virus season

For each of the 12 scenarios described in **Appendix Table 1**, **2**, and **3**, we run 200 stochastic simulations of the nine-month period between September 1, 2023 and April 23, 2024 and generate the following summary outputs at the US scale: (i) the median and 95% prediction intervals for daily cases, hospitalizations, and deaths, (ii) the cumulative numbers of cases, hospitalizations, and deaths over the prediction period, and (iii) the median and 95% prediction intervals for the timing of the peak, the magnitude of the peak.

We also analyzed four tripledemic (combined) scenarios (**Appendix Table 4**). For each of tripledemic scenarios, we generated 200 stochastic projections by adding together individual time series projections for each of the three pathogens (from the corresponding scenario groups). The

most optimistic tripledemic scenario (TRI-A) combines the higher medical countermeasures and lower transmissibility scenarios for each of the three pathogens: (i) a universal SARS-CoV-2 booster recommendation paired with no new SARS-CoV-2 variant (COV-A), (ii) higher influenza vaccine uptake paired with an H1N1 dominant season (INF-A), (iii) RSV vaccines recommended for older adults and RSV monoclonal antibodies recommended for all infants paired with pre-pandemic RSV transmission rate (RSV-A). The most pessimistic tripledemic scenario (TRI-D) combines the lower medical countermeasures and higher transmissibility scenarios for each of the three pathogens: (i) SARS-CoV-2 boosters recommended only for older adults paired with the emergence of a new SARS-CoV-2 variant (COV-A), (ii) lower influenza vaccine uptake paired with an H3N2 dominant season (INF-A), (iii) RSV vaccines not recommended and RSV monoclonal antibodies recommended only for high-risk infants paired with the higher 2022-2023 RSV transmission rate (RSV-A). We also consider two intermediate scenarios which combine the lower medical countermeasure and lower transmissibility scenarios for each of the three viruses (TRI-B) and higher medical countermeasure and higher transmissibility scenarios for each of the three viruses (TRI-C).

Supplementary Material B: Material and Methods

B.1 SARS-CoV-2 Model

We developed an age-structured SEIRS model of SARS-CoV-2 transmission that explicitly considers the XBB and EG.5 variants and SARS-CoV-2 original dose, booster, and reformulated booster vaccination[24] (**Appendix Figure 2**). The population-wide susceptibility to infection and severe disease (hospitalization) both depend on the circulating variants, immunity from the past infections and vaccinations, and rates of immune waning. Specifically, for each of the XBB and EG.5 variants, we include non-dimensional state variables that track population-wide protection against infection and severe disease[20]. For an age group, *a*, the changing levels of infection-derived protection against infection are given by:

$$\frac{dM^I_{C,a,EG}(t)}{dt} = \frac{k_C p_{EG}(t) R_a(t)}{N_a(1 + K_s \cdot \mathbf{M^I})} - \omega_C M^I_{C,a,EG}(t)$$

$$\frac{dM^I_{C,a,XB}(t)}{dt} = \frac{k_C p_{XB}(t) R_a(t)}{N_a(1 + K_s \cdot \mathbf{M^I})} - \omega_C M^I_{C,a,XB}(t)$$

where $M^I_{C,a,EG}(t)$ and $M^I_{C,a,XB}(t)$ are the population-level immunity against SARS-CoV-2 infection in age group *a* resulting from prior EG.5 and XBB infections, respectively. $p_{EG}(t)$ and $p_{XB}(t)$ denote the changing relative frequency of EG.5 and XBB across all infections, estimated by fitting logistic curves to variant proportion data[4] (**Appendix Figure 1**). Our model tracks the total amount of immunity in the population, which increases by a factor of k_C for each case that recovers and then wanes at a rate of ω_C. N_a is the total population of age group a, $R_a(t)$ denotes the number of recovered individuals in age group a; K_s is a positive constant modeling the saturation of antibody production in individuals who were previously infected and developed severe disease; ω_C represents the rate of waning of infection-derived immunity.

The changing levels of vaccine-derived protection against infection are given by:

$$\frac{dM^I_{C,a,V}(t)}{dt} = k_V V(t-\tau_V) - \omega_V M^I_{C,a,V}(t)$$

$$\frac{dM^I_{C,a,B}(t)}{dt} = k_B B(t-\tau_B) - \omega_B M^I_{C,a,B}(t)$$

$$\frac{dM^I_{C,a,RB}(t)}{dt} = k_{RB} RB(t-\tau_{RB}) - \omega_{RB} M^I_{C,a,RB}(t)$$

where $M^I_{C,a,V}(t)$, $M^I_{C,a,B}(t)$ and $M^I_{C,a,RB}(t)$ represent population-level immunity in age groups derived from primary, booster, and reformulated booster vaccines, respectively. $V(t)$, $B(t)$ and $RB(t)$ are the number of primary, booster, and reformulated boosters administered on day t, respectively, assuming that the administration of doses of primary vaccines, booster, or reformulated booster is delayed by τ_V, τ_B and τ_{RB} days respectively. The total amount of immunity in the population increases by a factor of k_V for each dose of primary vaccine administered and k_B , k_{RB} for each booster, reformulated booster administered, and then wanes at rates of ω_V and ω_B, ω_{RB} respectively. Population-level protection against hospitalization is similarly given by:

$$\frac{dM^H_{C,a,EG}(t)}{dt} = \frac{k_C p_{EG}(t) R_a(t)}{N_a(1+K_s \cdot \mathbf{M^H})} - \omega'_C M^H_{C,a,EG}(t)$$

$$\frac{dM^H_{C,a,XB}(t)}{dt} = \frac{k_C p_{XB}(t) R_a}{N_a(1+K_s \cdot \mathbf{M^H})} - \omega'_C M^H_{C,a,XB}(t)$$

$$\frac{dM^H_{C,a,V}(t)}{dt} = k_V V(t-\tau_V) - \omega'_V M^H_{C,a,V}(t)$$

$$\frac{dM^H_{C,a,B}(t)}{dt} = k_B B(t-\tau_B) - \omega'_B M^H_{C,a,B}(t)$$

$$\frac{dM^H_{C,a,RB}(t)}{dt} = k_{RB} RB(t-\tau_{RB}) - \omega'_{RB} M^H_{C,a,RB}(t)$$

where $M^H_{C,a,EG}(t)$, $M^H_{C,a,XB}(t)$, $M^H_{C,a,V}(t)$, $M^H_{C,a,B}(t)$ and $M^H_{C,a,RB}(t)$ represent population-level immunity against hospitalization derived from EG.5 infections, XBB infections, primary vaccines, booster vaccines, and reformulated booster vaccines, respectively. ω'_C denotes the rate of waning of population-level infection-acquired immunity against hospitalization. ω'_B, ω'_V, and ω'_{RB} denote the rates at which the immunity wanes for vaccine, booster and reformulated booster doses administered.

The average level of protection against infection in each age group is calculated by considering (i) the immune escape of circulating variants, (ii) the levels of immunity gained from infections and vaccinations, (iii) the severity of new infections, and (iv) the protection offered by each type of immunity against new infections. The immunity variables modify the transitions among disease compartments as given by:

$$\frac{dS_a(t)}{dt} = -S_a \cdot \sum_{i \in A} \frac{\beta_0 \phi_{a,i}(t)(I^Y_i(t) + I^A_i(t)\theta + P^Y_i(t) + P^A_i(t)\theta)}{N_i(1+\mathbf{K^I}(\mathbf{p}) \cdot \mathbf{M^I})} + \eta R_a(t)$$

$$\frac{dE_a(t)}{dt} = S_a \cdot \sum_{i \in A} \frac{\beta_0 \phi_{a,i}(t)(I_i^Y(t) + I_i^A(t)\theta + P_i^Y(t) + P_i^A(t)\theta)}{N_i(1 + \mathbf{K^I}(\mathbf{p}) \cdot \mathbf{M^I})} - \sigma E_a(t)$$

$$\frac{dP_a^A(t)}{dt} = (1 - \tau_l + \mathbf{K^I}(\mathbf{p}) \cdot \mathbf{M^I})\sigma E_a(t) - \rho^A P_a^A(t)$$

$$\frac{dP_a^Y(t)}{dt} = (\tau_l - \mathbf{K^I}(\mathbf{p}) \cdot \mathbf{M^I})\sigma E_a(t) - \rho^Y P_a^Y(t)$$

$$\frac{dI_a^A(t)}{dt} = \rho^A P_a^A(t) - \gamma^A I_a^A(t)$$

$$\frac{dI_a^Y(t)}{dt} = \rho^Y P_a^Y(t) - (1 - \mu_a)\gamma^Y I_a^Y(t) - \frac{\zeta \mu_a I_a^Y(t)}{1 + \mathbf{K^H}(\mathbf{p}) \cdot \mathbf{M^H}}$$

$$\frac{dH_a(t)}{dt} = \frac{\zeta \mu_a I_a^Y(t)}{1 + \mathbf{K^H}(\mathbf{p}) \cdot \mathbf{M^H}} - (1 - \nu_a)\gamma^H H_a(t) - \frac{\pi \nu_a H_a(t)}{1 + \mathbf{K^D}(\mathbf{p}) \cdot \mathbf{M^H}}$$

$$\frac{dR_a(t)}{dt} = \gamma^A I_a^A(t) + (1 - \mu_a)\gamma^Y I_a^Y(t) + (1 - \nu_a)\gamma^H H_a(t) - \eta R_a(t)$$

$$\frac{dD_a(t)}{dt} = \frac{\pi \nu_a H_a(t)}{1 + \mathbf{K^D}(\mathbf{p}) \cdot \mathbf{M^H}}$$

where $S_a(t)$, $E_a(t)$, $P_a^Y(t)$, $P_a^A(t)$, $I_a^Y(t)$, $I_a^A(t)$, $H_a(t)$, $R_a(t)$, and $D_a(t)$ are the age-specific numbers of people who are in the susceptible, exposed, pre-symptomatic, pre-asymptomatic, infectious symptomatic, infectious asymptomatic, hospitalized, recovered and death compartments, respectively. β_0 is the transmission rate. θ is the relative infectiousness of the compartments P_a^A I_a^A. The mixing rates between age groups a and i, $\phi_{a,i}(t)$, are based on published contact matrices (see below). The transition rates γ^A, γ^Y, γ^H denote the recovery rates for the I_a^Y, I_a^A, H_a compartments, respectively. ρ^A and ρ^Y denote the transition rates from pre-asymptomatic and pre-symptomatic to asymptomatic and symptomatic infections, respectively. σ denotes the transition rate out of exposed compartment, τ_l is the proportion of infections that are symptomatic, μ_a is the infection hospitalization rate, ζ is the transition rate from I_a^Y to H_a, ν_a is the in-hospital mortality rate, π is the transition rate from H_a to D_a, and η is the rate at which recovered individuals become susceptible again. N_i is the total population of the age group i. The vectors $\mathbf{K^I}(\mathbf{p}) = [K_{C,a,EG}^I(p), K_{C,a,XB}^I(p), K_{C,a,V}^I(p), K_{C,a,B(p)}^I, K_{C,a,RB}^I(p)]$, $\mathbf{K^H}(\mathbf{p}) = [K_{C,a,EG}^H(p), K_{C,a,XB}^H(p), K_{C,a,V}^H(p), K_{C,a,B(p)}^H, K_{C,a,RB}^H(p)]$, and $\mathbf{K^D}(\mathbf{p}) = [K_{C,a,EG}^D(p), K_{C,a,XB}^D(p), K_{C,a,V}^D(p), K_{C,a,B(p)}^D, K_{C,a,RB}^D(p)]$ contain positive constants that describe efficacy of immunity in reducing the rate of COVID-19 infection, hospitalization, and death, respectively. As described above, $\mathbf{M^I} = [M_{C,a,EG}^I, M_{C,a,XB}^I, M_{C,a,V}^I, M_{C,a,B}^I, M_{C,a,RB}^I]$ and $\mathbf{M^H} = [M_{C,a,EG}^H, M_{C,a,XB}^H, M_{C,a,V}^H, M_{C,a,B}^H, M_{C,a,RB}^H]$ are two vectors consisting of state variables that describe the protection levels derived from natural infection, vaccination, boosters and reformulated boosters against infection and hospitalization, respectively. We assume published estimates for rates of immune waning and that immunity against hospitalization wanes slower than immunity against infection[21,22]. The details of the COVID-19 model parameter values are summarized in **Appendix Table 5**.

B.2 Influenza Model

We use an age-structured SEIRS model of influenza transmission. Like the SARS-CoV-2 model described above, it explicitly tracks the immunity resulting from infection by either the influenza A(H1N1) or influenza A(H3N2) subtype and vaccination (**Appendix Figure 3**). The model tracks both *interseasonal immunity* that decreases rapidly at the onset of a new season and *intraseasonal immunity* that increases with new infections and vaccinations. For age group a, the changing levels of infection-derived protection against infection are given by:

$$\frac{dM^I_{F,a,H1}}{dt} = \frac{k_{inf} p_{H1}(t) R_a(t)}{N_a(1 + K_s \cdot \mathbf{M^I})} - \omega_{inf} M^I_{F,a,H1}(t)$$

$$\frac{dM^I_{F,a,H3}}{dt} = \frac{k_{inf} p_{H3}(t) R_a(t)}{N_a(1 + K_s \cdot \mathbf{M^I})} - \omega_{inf} M^I_{F,a,H3}(t)$$

where $M^I_{F,a,H1}(t)$ and $M^I_{F,a,H3}(t)$ denote population-level immunity resulting from H1N1 infections and H3N2 infections, respectively, in age group a. N_a is the total population of age group a, $R_a(t)$ denotes the number of recovered individuals in age group a. $p_{H1}(t)$ and $p_{H3}(t)$denote the changing prevalence of H1N1 and H3N2 across all infections. However, since both the subtypes circulate in most seasons, these values are set to 1 and 0 throughout the season depending on the subtype dominant season, where the value 1 corresponds to prevalence of the dominant strain.

The model tracks the total immunity in the population, which increases by a factor of k_{inf} for each case that recovers and wanes at a rate of ω_{inf}. K_s is a positive constant modeling the saturation of antibody production in individuals who were previously infected. The changing levels of vaccine-derived protection against infection are given by:

$$\frac{dM^I_{F,a,V}(t)}{dt} = k_F V(t - \tau_F) - \omega_F M^I_{F,a,V}(t)$$

where $M^I_{F,a,V}(t)$ represents population-level immunity derived from vaccines, respectively. $V(t)$ is the number of vaccine doses administered at time t and τ_F represents the delay in the number of days of dose administration. The total immunity in the population increases by a factor of k_F for each dose of vaccine administered and then wanes at rates of ω_F. Similarly, we describe the changing levels of population immunity against hospitalization as given by:

$$\frac{dM^H_{F,a,H1}(t)}{dt} = \frac{k_{inf} p_{H1}(t) R_a(t)}{N_a(1 + K_s \cdot \mathbf{M^H})} - \omega'_{inf} M^H_{F,a,H1}(t)$$

$$\frac{dM^H_{F,a,H3}(t)}{dt} = \frac{k_{inf} p_{H3}(t) R_a(t)}{N_a(1 + K_s \cdot \mathbf{M^H})} - \omega'_{inf} M^H_{F,a,H3}(t)$$

$$\frac{dM^H_{F,a,V}(t)}{dt} = k_F V(t - \tau_F) - \omega'_F M^H_{F,a,V}(t)$$

where $M^H_{F,a,H1}$, $M^H_{F,a,H3}$ and $M^H_{F,a,V}$ represent population-level immunity against influenza hospitalizations derived from infections by the H1N1 subtype, infections by the H3N2 subtype, and vaccines, respectively. ω'_{inf} denotes the rate of waning of population-level immunity against hospitalization and ω'_F denotes the rate at which the immunity wanes for vaccine dose administered.

These immunity variables modify the age-specific transitions among disease compartments as given by:

$$\frac{dS_a(t)}{dt} = -S_a(t) \cdot \sum_{i \in A} \frac{\beta(t)\phi_{a,i}(t)I_i(t)}{N_i(1 + \mathbf{K^{I_F}}(\mathbf{p}) \cdot \mathbf{M^{I_F}})} + \eta R_a(t)$$

$$\frac{dE_a(t)}{dt} = S_a(t) \cdot \sum_{i \in A} \frac{\beta(t)\phi_{a,i}(t)I_i(t)}{N_i(1 + \mathbf{K^{I_F}}(\mathbf{p}) \cdot \mathbf{M^{I_F}})} - \sigma E_a(t)$$

$$\frac{dI_a(t)}{dt} = \sigma E_a(t) - (1 - \mu_a)\gamma I_a(t) - \frac{\zeta \mu_a I_a(t)}{1 + \mathbf{K^{H_F}}(\mathbf{p}) \cdot \mathbf{M^{H_F}}}$$

$$\frac{dH_a(t)}{dt} = \frac{\zeta \mu_a I_a}{1 + \mathbf{K^{H_F}}(\mathbf{p}) \cdot \mathbf{M^{H_F}}} - (1 - \nu_a)\gamma_H H_a(t) - \frac{\pi \nu_a H_a(t)}{1 + \mathbf{K^{D_F}}(\mathbf{p}) \cdot \mathbf{M^{H_F}}}$$

$$\frac{dR_a(t)}{dt} = (1 - \mu_a)\gamma I_a(t) + (1 - \nu_a)\gamma_H H_a(t) - \eta R_a(t)$$

$$\frac{dD_a(t)}{dt} = \frac{\pi \nu_a H_a(t)}{1 + \mathbf{K^{D_F}}(\mathbf{p}) \cdot \mathbf{M^{H_F}}}$$

where $S_a(t)$, $E_a(t)$, $I_a(t)$, $H_a(t)$ and $R_a(t)$ are age-specific numbers of people who are in the susceptible, exposed, infectious, hospitalized, recovered and death compartments, respectively, at time t. The time-dependent transmission rate is given by $\beta(t) = \beta_0(1 + q(t))$, where β_0 is the transmission rate and $q(t)$ is a seasonality parameter based on absolute humidity (see below). The mixing rates between age groups a and i, $\phi_{a,i}(t)$, are based on published contact matrices (see below). The transition parameters γ, γ_H denote the recovery rates for the $I_a(t)$ and $H_a(t)$ compartments, respectively, σ denotes the transition rate out of exposed compartment, μ_a is the infection hospitalization rate, ζ is the transition rate from I_a to H_a, ν_a is the in-hospital mortality rate, π is the transition rate from H_a to D_a, and η is the rate at which recovered individuals become susceptible again. N_i is the total population of the age group i. The vectors $\mathbf{K^{I_F}}(\mathbf{p}) = [K^I_{F,a,H1}(p), K^I_{F,a,H3}(p), K^I_{F,a,V}(p)]$, $\mathbf{K^{H_F}}(\mathbf{p}) = [K^H_{F,a,H1}(p), K^H_{F,a,H3}(p), K^H_{F,a,V}(p)]$ and $\mathbf{K^{D_F}}(\mathbf{p}) = [K^D_{F,a,H1}(p), K^D_{F,a,H3}(p), K^D_{F,a,V}(p)]$ contain positive constants that describe efficacy of immunity in reducing the rate of influenza infection, hospitalization, and death, respectively. As described above, $\mathbf{M^{I_F}} = [M^I_{F,a,H1}, M^I_{F,a,H3}, M^I_{F,a,V}]$ and $\mathbf{M^{H_F}} = [M^H_{F,a,H1}, M^H_{F,a,H3}, M^H_{F,a,V}]$ are two vectors consisting of state variables that describe the protection levels derived from natural infection and vaccination against infection and hospitalization, respectively. The overall structure of the model is similar to that of the COVID-19 model, with the exception of influenza subtypes, vaccination, and seasonality features. The details of the Influenza model parameter values are summarized in **Appendix Table 6**.

B.3 Respiratory Syncytial Virus (RSV) Model

As for SARS-CoV-2 and influenza, we model the transmission dynamics of RSV in US using an age-structured stochastic compartmental SEIRS model that explicitly tracks immunity resulting from natural infections, monoclonal antibodies for infants, and vaccination for adults over age 60 years (**Appendix Figure 4**). Rather than explicitly modeling the vaccination of pregnant women, our high intervention scenarios assume that infants are born with passive immunity against RSV

acquired in utero. For the age group a, the changing levels of infection-derived protection against infection are given by:

$$\frac{dM^{I}_{R,a,\iota(t)}}{dt} = \frac{k_\iota R_a(t)}{N_a(1 + K_s \cdot \mathbf{M^{I_R}})} - \omega_\iota M^{I}_{R,a,\iota(t)}$$

where $M^{I}_{R,a,\iota}(t)$ is model population-level immunity resulting from RSV infections in age group a. N_a is the total population of age group a. $R_a(t)$ denotes the number of recovered individuals in age group a. The model tracks the total amount of immunity in the population, which increases by a factor of k_ι for each case that recovers and then wanes at a rate of ω_ι. K_s is a positive constant modeling the saturation of antibody production in individuals who were previously infected. The changing levels of protection against infection derived from vaccines and monoclonal antibodies are given by:

$$\frac{dM^{I}_{R,a,V}(t)}{dt} = k_R V(t - \tau_R) - \omega_R M^{I}_{R,a,V}(t)$$

$$\frac{dM^{I}_{R,a,M_A}(t)}{dt} = k_R M_A(t) - \omega_R M^{I}_{R,a,M_A}(t)$$

where $M^{I}_{R,a,V}(t)$ and $M^{I}_{R,a,M_A}(t)$ represents population-level immunity derived from vaccines and monoclonal antibodies respectively. $V(t)$ is number of vaccine doses administered on day t and τ_R represents the delay in the number of days of vaccine dose administered. For the monoclonal antibodies there is no delay considered and $M_A(t)$ represents the number of monoclonal antibodies administered. The total amount of immunity in the population increases by a factor of k_R for each dose of vaccine or monoclonal antibodies administered and then wanes at rates of ω_R. Similarly, we describe the changes in the population-level immunity against hospitalization as given by:

$$\frac{dM^{H}_{R,a,\iota}(t)}{dt} = \frac{k_\iota R_a(t)}{N_a(1 + K_s \cdot \mathbf{M^{H_R}})} - \omega'_\iota M^{H}_{R,a,\iota}(t)$$

$$\frac{dM^{H}_{R,a,V}(t)}{dt} = k_R V(t - \tau_R) - \omega'_R M^{H}_{R,a,V}(t)$$

$$\frac{dM^{H}_{R,a,M_A}(t)}{dt} = k_R M_A(t) - \omega'_R M^{H}_{R,a,M_A}(t)$$

where $M^{H}_{R,a,\iota}(t)$, $M^{H}_{R,a,V}(t)$ and $M^{H}_{R,a,M_A}(t)$ represent population-level immunity against hospitalization derived from RSV infections, vaccines, and monoclonal antibodies, respectively. N_a is the total population of the age group a. ω'_ι denotes the rate of waning of population-level immunity against hospitalization due to natural infection and ω'_R denotes the rate at which the immunity wanes for vaccine/monoclonal antibody dose administered. The immunity variables modify the transitions among disease compartments as given by:

$$\frac{dS_a(t)}{dt} = -S_a(t) \cdot \sum_{i \in A} \frac{\beta(t)\phi_{a,i}(t)I_i(t)}{N_i(1 + \mathbf{K^{I_R}} \cdot \mathbf{M^{I_R}})} + \eta R_a(t)$$

$$\frac{dE_a(t)}{dt} = S_a(t) \cdot \sum_{i \in A} \frac{\beta(t)\phi_{a,i}(t)I_i(t)}{N_i(1 + \mathbf{K^{I_R}} \cdot \mathbf{M^{I_R}})} - \sigma E_a(t)$$

$$\frac{dI_a(t)}{dt} = \sigma E_a(t) - (1-\mu_a)\gamma I_a(t) - \frac{\zeta \mu_a I_a(t)}{1 + \mathbf{K^{H_R}} \cdot \mathbf{M^{H_R}}}$$

$$\frac{dH_a(t)}{dt} = \frac{\zeta \mu_a I_a(t)}{1 + \mathbf{K^{H_R}} \cdot \mathbf{M^{H_R}}} - (1-\nu_a)\gamma_H H_a(t) - \frac{\pi \nu_a H_a(t)}{1 + \mathbf{K^{D_R}} \cdot \mathbf{M^{H_R}}}$$

$$\frac{dR_a(t)}{dt} = (1-\mu_a)\gamma I_a(t) + (1-\nu_a)\gamma_H H_a(t) - \eta R_a(t)$$

$$\frac{dD_a(t)}{dt} = \frac{\pi \nu_a H_a(t)}{1 + \mathbf{K^{D_R}} \cdot \mathbf{M^{H_R}}}$$

where $S_a(t)$, $E_a(t)$, $I_a(t)$, $H_a(t)$, $R_a(t)$, and $D_a(t)$ are the age-specific numbers of people who are in the susceptible, exposed, infectious, hospitalized, recovered and death compartments, respectively, at time t. The time dependent transmission rate is given by $\beta(t) = \beta_0(1 + q(t))$, where β_0 is the transmission rate, $q(t)$ is the seasonality parameter based on absolute humidity (see below). The mixing rates between age groups a and i, $\phi_{a,i}(t)$, are based on published contact matrices (see below). The transition parameters γ, γ_H denote the recovery rates for the I_a and H_a compartments, respectively, σ denotes the transition rate out of exposed compartment, μ_a is the infection hospitalization rate, ζ is the transition rate from I_a to H_a, ν_a is the in-hospital mortality rate, π is the transition rate from H_a to D_a, and η is the rate at which recovered individuals become susceptible again. N_i is the total population of the age group i. The vectors $\mathbf{K^{I_R}} = [K^I_{R,a,\iota}, K^I_{R,a,V}, K^I_{R,a,M_A}]$, $\mathbf{K^{H_R}} = [K^H_{R,a,\iota}, K^H_{R,a,V}, K^H_{R,a,M_A}]$ and $\mathbf{K^{D_R}} = [K^D_{R,a,\iota}, K^D_{R,a,V}, K^D_{R,a,M_A}]$ contain positive constants that describe efficacy of immunity in reducing the rate of RSV infection, hospitalization, and death, respectively. As described above, $\mathbf{M^{I_R}} = [M^I_{R,a,\iota}, M^I_{R,a,V}, M^I_{R,a,M_A}]$ and $\mathbf{M^{H_R}} = [M^H_{R,a,\iota}, M^H_{R,a,V}, M^H_{R,a,M_A}]$ are two vectors consisting of state variables that describe the protection levels derived from natural infection, vaccination and monoclonal antibodies against infection and hospitalization, respectively. The overall structure of the model is similar to that of the COVID-19 model, with the exception of RSV vaccination, monoclonal antibody immunization and seasonality features. The details of the RSV model parameter values are summarized in **Appendix Table 7**.

B.4 Incorporating parameter uncertainty and stochastic dynamics

We incorporate stochasticity into our projections of SARS-CoV-2, influenza, and RSV transmissions by both varying the daily transmission rate β_0 and introducing variation into the transition rates between disease compartments. For each day, we draw a random value from the estimated normal distribution of β_0 values (**Appendix Table 5, 6 and 7**). We introduce stochasticity into the disease progression models using the tau-leap method[27], in which the transition rates are sampled from Poisson distribution with mean equal to the deterministic rates multiplied by the time step τ. In addition to this in the case of RSV, stochasticity is included in the immunity against infection by introducing variation into the initial estimates of the age specific - immunity parameter by drawing a random value from the estimated normal distribution of initial immunity.

For example, the stochastic versions our SARS-CoV-2 model is given by:

$$dS_a(t+\tau) = \text{Pois}\left(-\tau S_a \cdot \sum_{i \in A} \frac{\beta_0 \phi_{a,i}(t)(I_i^Y(t) + I_i^A(t)\theta + P_i^Y(t) + P_i^A(t)\theta)}{N_i(1 + \mathbf{K^I}(\mathbf{p}) \cdot \mathbf{M^I})}\right) + \text{Pois}(\tau\eta R_a(t))$$

$$dE_a(t+\tau) = \text{Pois}\left(\tau S_a \cdot \sum_{i} \frac{\beta_0 \phi_{a,i}(t)(I_i^Y(t) + I_i^A(t)\theta + P_i^Y(t) + P_i^A(t)\theta)}{N_i(1 + \mathbf{K^I}(\mathbf{p}) \cdot \mathbf{M^I})}\right) - \text{Pois}(\tau\sigma E_a(t))$$

$$dP_a^A(t+\tau) = \text{Pois}\left(\tau(1 - \tau_l + \mathbf{K^I}(\mathbf{p}) \cdot \mathbf{M^I})\sigma E_a(t)\right) - \text{Pois}(\tau\rho^A P_a^A(t))$$

$$dP_a^Y(t+\tau) = \text{Pois}\left(\tau(\tau_l - \mathbf{K^I}(\mathbf{p}) \cdot \mathbf{M}(\mathbf{I}))\sigma E_a(t)\right) - \text{Pois}(\tau\rho^Y P_a^Y(t))$$

$$dI_a^A(t+\tau) = \text{Pois}(\tau\rho^A P_a^A(t)) - \text{Pois}(\tau\gamma^A I_a^A(t))$$

$$dI_a^Y(t+\tau) = \text{Pois}(\tau\rho^Y P_a^Y(t)) - \text{Pois}(\tau(1-\mu_a)\gamma^Y I_a^Y(t)) - \text{Pois}\left(\frac{\tau\zeta\mu_a I_a^Y(t)}{1 + \mathbf{K^H}(\mathbf{p}) \cdot \mathbf{M^H}}\right)$$

$$dH_a(t+\tau) = \text{Pois}\left(\frac{\tau\zeta\mu_a I_a^Y(t)}{1 + \mathbf{K^H}(\mathbf{p}) \cdot \mathbf{M^H}}\right) - \text{Pois}(\tau(1-\nu_a)\gamma^H H_a(t)) - \text{Pois}\left(\frac{\tau\pi\nu_a H_a(t)}{1 + \mathbf{K^D}(\mathbf{p}) \cdot \mathbf{M^H}}\right)$$

$$dR_a(t+\tau) = \text{Pois}(\tau\gamma^A I_a^A(t)) + \text{Pois}(\tau(1-\mu_a)\gamma^Y I_a^Y(t)) + \text{Pois}(\tau(1-\nu_a)\gamma^H H_a(t)) - \text{Pois}(\tau\eta R_a(t))$$

$$dD_a(t+\tau) = \text{Pois}\left(\frac{\tau\pi\nu_a H_a(t)}{1 + \mathbf{K^D}(\mathbf{p}) \cdot \mathbf{M^H}}\right)$$

The stochastic versions of our influenza and RSV transmission models are similarly adapted from the deterministic equations (not shown).

For each scenario projection, we run 200 stochastic simulations. We compute the seven-day rolling averages of projected hospitalizations and deaths and summarize their evolving distributions using the 0.025, 0.50, and 0.975 quantiles for each day (parameters are included in **Table B.1** to **B.3**).

B.5 Age-specific contact patterns

We model daily age-specific mixing patterns using published estimates for venue-specific (all locations, school, and work) contact rates in the US[28]. We assume that schools close on all weekends, during a two week winter break (December 18 to January 02), and throughout the summer break (June-August). Workplaces are assumed to be closed during the weekends. The overall contact matrix on day *t* is given by:

$$\phi_{i,a}(t) = C_{\text{all}} - \alpha_s(t)C_s - \alpha_w(t)C_w,$$

where C_{all}, C_s *and* C_w are the estimated age-specific contact matrices for all locations, schools, and workplaces, respectively. $\alpha_s(t)$ *and* $\alpha_w(t)$ are time-dependent functions that describe the opening or closure of schools and workplaces, they equal 0 if the location is closed and 1 if it is closed. For the SARS-CoV-2 and influenza models, we consider six age groups [0-4, 5-11, 12-18, 19-49, 50-64, 65+] and assume the following contact matrices:

$$C_{\text{all}} = \begin{bmatrix} 2.598237 & 1.600682 & 0.1895988 & 4.1198752 & 0.912514 & 0.112739 \\ 0.640235268 & 8.428533343 & 0.400015072 & 4.028603965 & 0.709643468 & 0.103204179 \\ 0.173684 & 2.0999574 & 6.663684 & 8.710766 & 0.5601588 & 0.0327582 \\ 0.490443671 & 1.516968944 & 0.759891199 & 10.27014274 & 1.714438659 & 0.095919246 \\ 0.431143971 & 1.339346998 & 0.592373724 & 6.379632659 & 3.196133287 & 0.188612431 \\ 0.204998347 & 0.718001781 & 0.182731115 & 2.136319698 & 1.558267141 & 0.602532372 \end{bmatrix},$$

$$C_s = \begin{bmatrix} 1.196597632 & 0.269627261 & 0.03173379 & 0.38262616 & 0.049755762 & 0 \\ 0.139739606 & 3.973684579 & 0.051319078 & 0.369792419 & 0.075075384 & 0.000263253 \\ 0.016961126 & 0.903246574 & 3.427856164 & 2.582830513 & 0.060321191 & 0 \\ 0.058180033 & 0.331477088 & 0.188215674 & 0.461408137 & 0.042344186 & 0.000352703 \\ 0.093904827 & 0.568170143 & 0.243358213 & 0.35953993 & 0.073783363 & 0.0005338 \\ 0.000729122 & 0.021954765 & 0.006167126 & 0.029787663 & 0.03474166 & 0.011651215 \end{bmatrix},$$

$$C_w = \begin{bmatrix} 0 & 0 & 0 & 0 & 0 & 1.20585 \times 10^{-05} \\ 0 & 0.039768604 & 0.005775822 & 0.091897952 & 0.006139445 & 0 \\ 0 & 0.020170591 & 0.386451333 & 1.666005478 & 0.136647372 & 0 \\ 0 & 0.056904943 & 0.171469933 & 4.893999929 & 0.792456512 & 0 \\ 0 & 0.069619305 & 0.071928236 & 2.526315884 & 0.70871039 & 0 \\ 0 & 0 & 0 & 0.00026916 & 8.88673 \times 10^{-05} & 2.02847 \times 10^{-05} \end{bmatrix}.$$

For the RSV model, we separate young children into 0-1 and 2-4 age brackets for a total of seven age groups ([0-1, 2-4, 5-11, 12-18, 19-49, 50-64, 65+]) and assume the following contact matrices:

$$C_{\text{all}} = \begin{bmatrix} 0.41571792 & 0.62357688 & 0.6402728 & 0.07583952 & 1.64795008 & 0.3650056 & 0.0450956 \\ 0.62357688 & 0.93536532 & 0.9604092 & 0.11375928 & 2.47192512 & 0.5475084 & 0.0676434 \\ 0.256063698 & 0.384095547 & 8.428928856 & 0.400056795 & 4.028610697 & 0.709638833 & 0.103207004 \\ 0.0694736 & 0.1042104 & 2.0999574 & 6.663684 & 8.710766 & 0.5601588 & 0.0327582 \\ 0.196444181 & 0.294666271 & 1.536702073 & 0.75586858 & 10.23993044 & 1.716071999 & 0.096681476 \\ 0.172857814 & 0.25928672 & 1.316125809 & 0.580376819 & 6.277775206 & 3.177097547 & 0.19167384 \\ 0.08146504 & 0.12219756 & 0.713464135 & 0.186643907 & 2.06378641 & 1.461248924 & 0.617200751 \end{bmatrix}$$

$$C_w = \begin{bmatrix} 0.22426224 & 0.33639336 & 0.532422 & 0.06314592 & 1.49489968 & 0.3451032 & 0.0450956 \\ 0.33639336 & 0.50459004 & 0.798633 & 0.09471888 & 2.24234952 & 0.5176548 & 0.0676434 \\ 0.200180313 & 0.30027047 & 4.455040339 & 0.348729456 & 3.65881409 & 0.634561526 & 0.102943726 \\ 0.0626892 & 0.0940338 & 1.1967108 & 3.2358276 & 6.127936 & 0.4998384 & 0.0327582 \\ 0.173040012 & 0.259560017 & 1.204009618 & 0.56839856 & 9.783531219 & 1.673878384 & 0.096356199 \\ 0.135563906 & 0.203345858 & 0.766610009 & 0.342050483 & 5.92489155 & 3.103818202 & 0.191159289 \\ 0.081231636 & 0.121847455 & 0.692157683 & 0.179345556 & 2.036189571 & 1.431782451 & 0.607203661 \end{bmatrix}$$

B.6 Modeling seasonality

We incorporate humidity-based seasonality in our influenza and RSV models[29]. We collected the absolute humidity data from 2006 to 2020 at the National Oceanic and Atmospheric Administration (NOAA)[30]. We estimated the daily absolute humidity by averaging the collected data for the corresponding dates. We incorporate a seasonality parameter *q(t)* into the model equations as follows:

$$q(t) = \left(\frac{\text{avg}(ah) - ah(t)}{\max(ah) - \text{avg}(ah)} \right) \times \xi,$$

where ξ is the magnitude of the seasonality impact on transmissibility and $ah(t)$ is the estimated absolute humidity on calendar day t (based on the fitted cosine model), $\max(ah)$is the value of the maximum absolute humidity in a year, and $\text{avg}(ah)$ is the value of the average maximum absolute humidity in a year (**Figure 5**).

B.7 Data and model fitting

For each of the three virus, we iteratively calibrate the transmission rate β_0 and the age-specific infections to hospitalization rate μ_a using least squares fitting. We initially set the μ_a equal to published values for COVID-19[31], influenza[32], and RSV[33] , and then repeatedly (i) estimate β_0 by fitting the models to incident hospitalization time series data for COVID-19[34], influenza[34], and RSV[12] and (ii) estimate the μ_a by fitting the models to age-specific cumulative hospitalizations for COVID-19[34], influenza[35,36], and RSV[12]. We repeat this two step fitting process until projected distribution of incident hospitalization across age groups is within 2% of the observed distribution.

For estimating COVID-19, influenza and RSV transmission rates (β_0) we use a unified approach where a single calibration period is employed. For COVID-19 the fitting period spans from December 1, 2022 to July 11, 2023. In the case of influenza the fitting period spans from August 8, 2022 to July 5 2023 and for RSV, the fitting period is between October 3 2022 and July 18 2023. Additionally in the case of RSV low transmission scenario, the transmission rate is (β_0) is obtained by fitting the model for the periods between October 1 2018 and May 5 2019 and September 30 2019 and May 2 2020 and obtaining the mean transmission rate of the two periods, which is then again subjected to seasonal forcing when fitting to the 2022-2023 RSV season and projection phases. The projection period for all the three diseases ends by April 23 2024. In the case of influenza and RSV model, during the fitting and projection phases, the transmission rate (β_0) is subject to the effects of seasonality, which fluctuates over time and is factored into β_0 giving the time dependent transmission rate:

$$\beta(t) = \beta_0(1 + q(t))$$

The seasonality magnitude (see above) in the specific humidity parameter ($q(t)$) is estimated simultaneously along with the transmission rate. Additionally, for RSV, during the fitting process of the model to hospitalization data using the least squares method, the age specific level of initial immunity, alongside other parameters like transmission rate and seasonality magnitude , are calibrated simultaneously. The objective for revising the RSV model fitting method is to reflect the pre and post COVID impact, the decline in viral immunity in sensitive age groups, and the change in RSV epidemiology caused by the pandemic[37].

Finally, for COVID-19 and influenza, we calibrate the age-specific in-hospital mortality rate ν_a by first assuming that they are proportional to published estimates for in-hospital mortality rates for COVID-19[38] and influenza[39], and then scaling the rates by least squares fitting to reported age-specific mortality rates for COVID-19[40] and influenza[40]. For RSV, we assumed published estimates for ν_a[41].

B.8 Modeling immune escape for COVID-19

For COVID-19, we model immune escape by variants by reducing the values of parameters that govern the impact of population-level immunity on susceptibility and severity, as given by:

$$K^{I}_{C,a,j}(p) = M^{I}_{C,a,j}(1 - p\epsilon),$$

where j can be either XBB, EG.5, COVID-19 vaccines or boosters, $M^{I}_{C,a,j}(p)$ is the population-level immunity resulting from j, $K^{I}_{C,a,j}(p)$ represents the effective population-level immunity against the emerging new variant j resulting from the existed immunity $M^{I}_{C,a,j}(p)$, p is the relative prevalence of the emerging variant to the previous variant, ϵ represents the levels of immune escape to infection, and $(1 - p\epsilon)$ represents the efficacy of existing immunity against the emerging variant.

We consider that immunity acquired through infection with a specific variant provides the best protection against the same variant [73]. Also, we assume that all Omicron variants do not escape immunity acquired by booster shots [74]. The protection levels provided by vaccines and natural infection are included in **Appendix Table 5.**

B.9 Estimating initial levels of infection-acquired and vaccine-acquired immunity

For SARS-CoV-2, age-specific patterns for immunity $(M^{I}_{C,a,EG})$ and the immunity from the previous Omicron variants were assumed to match the data for seroprevalence[4] and hospitalization[34]. In particular, we use the available US seroprevalence data from January, 2022 to March, 2022 to estimate the value of k_C, which is the conversion rate of population immunization from natural infections. We then fit the model to COVID-19 Reported Patient Impact and Hospital Capacity by State Time Series hospitalization data from March 1, 2022 to August 31, 2022 to estimate the levels of population immunity on September 1, 2022, accounting for waning. To estimate the initial levels of vaccine- and booster-acquired immunity, we use the COVID-19 Vaccinations in the United States, jurisdiction data[75] from December 1st, 2021 to May 12, 2023, accounting for waning, to model the age-specific vaccine uptake before the projection period. The first date for vaccination is considered to be two weeks before the beginning of the model fitting for primary series, and one week for boosters. Vaccine-induced immunity was initiated by considering the vaccination coverages, in terms of administered doses per age group, until the starting date of the fitting period.

For influenza, age-specific patterns for initial immunity $(M^{I}_{F,a,H1})$ are estimated by using the fitted model to simulate infections during the 2022-2023 influenza season (August 8, 2022 - July 5, 2023), accounting for waning. To estimate the initial levels of vaccine-acquired immunity, we use the CDC Flu Vaccination Coverage data[76] from July 1, 2022 to May 31, 2023, accounting for waning, to model the age-specific vaccine uptake before the projection period. The first date for vaccination is considered to be two weeks before the beginning of the simulation for flu vaccines. Vaccine-induced immunity was initiated by considering the vaccination coverages, in terms of administered doses per age group, until the starting date of the fitting period.

For RSV, age-specific levels of initial immunity $(M^{I}_{R,a,\iota})$ are estimated by using the fitted model to simulate infections during the 2022-2023 RSV season (Oct 1, 2022 and July 18, 2023), accounting for waning. We assume that there is no vaccine- or monoclonal antibody-acquired immunity at the start of the simulations.

B.10 Modeling age-specific vaccination rates

SARS-CoV-2 vaccination is modeled by considering the daily number of allocated doses for both model fitting and projection periods. These doses can be either administered during primary series or as additional shots. We assume that each administered dose upregulates the age-specific immunity M_V^I two weeks after its administration. The number of administered doses per age group is based on taken from the CDC COVID-19 Vaccination and Case Trends by Age Group data[46]. For scenarios COV-B and COV-D, uptake of reformulated booster in the 65+ group follows uptake previously observed for the first booster dose authorized in September 2021[75](totaling 44% population coverage in age 65+ by). For scenarios COV-A and COV-C, uptake of reformulated booster in all eligible individuals is assumed to reach the level of coverage of the 2021 booster by February 1, 2024 (approximately 34% uptake nationally).

The assumed influenza vaccination patterns are based on coverage data from the 2022-23 influenza season in the US[76]. We simulate uptake during the 2022-2023 season based on the CDC Flu Vaccination Coverage data[76] administered by age group (totaling 51% population coverage). For the 2023-2024 projection period, we assume that the vaccines roll out at the same pace, scaled to reach either 40% or 60% total population coverage, depending on the scenario.

For RSV, the new vaccines[77] and monoclonal antibodies[78] were approved in mid 2023. Thus we assume no uptake of either mode of immunization prior to the start of the 2023-2024 projection period. The high intervention scenarios (RSV-A/RSV-C) assume that (i) RSV vaccines roll out to adults over 60y at the same pace that influenza vaccines were administered during the 2022-2023 season[76], reaching 56.12% of adults over 60y, (ii) all newborns are immediately immunized through maternal prepartum vaccination, and (iii) monoclonal antibodies roll out to infants under eight months linearly, reaching 100% coverage of children born before September 1, 2023 by December 31, 2023. The low intervention scenarios (RSV-B/RSV-D) assume that no adults receive vaccines and only high risk children under 1 years old receive monoclonal antibodies (approximately 1.7% uptake nationally)[79].

B.11 Modeling Validation

To validate our models, we typically fit a model to a 4-month to 9-month training period and then make projections for a 4-month to 10-month testing period. We compare the projected to the observed age-specific hospital admission data during the testing period in terms of the timing of peak hospitalizations, the magnitude of peak hospitalizations, and cumulative hospitalizations (**Appendix Table 8**).

The COVID-19 86model was previously developed, validated, and applied to provide projections for the US COVID-19 Scenario Modeling Hub, Rounds 12 to 17[23]. In addition, we have used the model to project the Omicron BA.1, BA.4, and BA.5 emergence in the US and Texas[24] . In this paper, we used our projections of the BA.4 and BA.5 emergence to validate our model.

The influenza model was previously developed, validated, and applied to provide projections for the US Influenza Scenario Modeling Hub, 2022/2023 Round 1 to 3, and 2023/2024 Round 1[26]. In this paper, we used our projections of the US Influenza Scenario Modeling Hub 2022/2023 Round 3 to validate our model.

In this paper, we built a new RSV model. We validated our RSV model by fitting it to RSV hospitalization data from the 2018-2019 season and then comparing projections for the 2019-2020 RSV season to reported RSV hospitalization data (**Appendix Figure 6**).

Supplementary material C: Summary of projection results and retrospective epidemiological trends

The results provided in this document are rounded to the nearest ones digit; the results in the main article are rounded to the nearest thousand.

Supplementary Material D: Comparison between historic and updated projections

In August 2023, we projected hospitalizations in the United States due to COVID-19, influenza, and RSV during the 2023-2024 respiratory virus season under plausible scenarios for viral transmission rates and the impacts of influenza vaccines, SARS-CoV-2 boosters, and newly-approved RSV vaccines and monoclonal antibodies[81]. Shortly after submitting our original projections to the CDC, we improved our methodology for modeling the humidity-driven seasonality of RSV and influenza and for estimating the (non-seasonal) transmission rate parameter for COVID, which we applied to create the projections reported in this article. The updated projections still use only data that were available during the summer of 2023. In comparing our original projections with those reported here, we find that the projected total hospitalizations is similar, but that the timing of the influenza and RSV epidemic peaks in the updated projections are more consistent with the subsequently observed data[81].

As additional model validation, we retrospectively analyzed an additional set of COVID, influenza, and RSV scenarios that more closely reflect the 2023-2024 season. As of April 2024, the 2023-2024 influenza season was dominated by type A H1N1, with small proportions of type A H3N2 and type B[84], and an estimated 47.3% of the US population received a seasonal influenza vaccine[85]. Our projections for this scenario, using a model fit to data through June 2023, are consistent with the observed epidemiological trends through February 2024 (**Appendix Figure 9**).

As of February 24, 2024, an estimated 43.0% (95% CI: 33.9%-52.1%) of infants under eight months in the US received Nirsevimab[19]; as of March 31, 2024, an estimated 23.6% (95% CI: 22.7%-24.5%) of adults over age 60 in the US received an RSV vaccine[25]. We also observed that the adult RSV hospitalization rate in 2023-2024 was significantly higher than the prior season[12], which may stem from behavior changes (e.g., fewer adults wearing face masks)[71]. Our projections for a scenario assuming the observed immunization rates and a 30% higher transmission rate in adults over age 18 provides a reasonable match with the eventual epidemiological trends (**Appendix Figure 10**).

We were unable to design a simple SARS-CoV-2 scenario that reproduced the two-wave structure of the 2023-2024 season, in which a modest autumn wave (dominated by the EG.5, HV.1, and FL

1.51 variants) preceded a larger winter wave (dominated by the JN.1 variant)[4]. In future work, we will consider additional complexities, including climate-driven seasonality[72] and variant-specific severity and rates of immune waning.

References

1. Ma KC, Shirk P, Lambrou AS, Hassell N, Zheng XY, Payne AB, et al. Genomic surveillance for SARS-CoV-2 variants: circulation of omicron lineages—United States, January 2022–May 2023. MMWR Morb Mortal Wkly Rep. 2023;72:651–6. PubMed https://doi.org/10.15585/mmwr.mm7224a2

2. World Health Organization. EG.5 initial risk evaluation, 9 August 2023 [cited 2023 Aug 18]. https://www. who. int/docs

3. Lin D-Y, Xu Y, Gu Y, Zeng D, Wheeler B, Young H, et al. Effectiveness of bivalent boosters against severe omicron infection. N Engl J Med. 2023;388:764–6. PubMed https://doi.org/10.1056/NEJMc2215471

4. Centers for Disease Control and Prevention. COVID data tracker. Centers for Disease Control and Prevention. 2020 [cited 2023 Aug 18]. https://covid.cdc.gov/covid-data-tracker

5. FluView Interactive. 2020 [cited 2024 Sep 4]. https://www.cdc.gov/flu/weekly/fluviewinteractive.htm

6. Australian Government Department of Health & Care. A. Australian Influenza Surveillance Reports. Australian Government Department of Health and Aged Care. 2023 [cited 2023 Aug 18]. https://www.health.gov.au/resources/collections/aisr?language=en

7. Estimated flu-related illnesses, medical visits, hospitalizations, and deaths in the United States — 2019–2020 flu season. 2023 [cited 2023 Aug 18]. https://www.cdc.gov/flu/about/burden/2019-2020.html

8. Kahn B, Brown L, Foege W, Gayle H, editors. Framework for equitable allocation of COVID-19 vaccine. Washington: National Academies Press (US); 2020.

9. Historical reference of seasonal influenza vaccine doses distributed. Centers for Disease Control and Prevention. 2020 [cited 2023 Aug 18]. https://www.cdc.gov/flu/hcp/vaccine-supply/vaccine-supply-historical.html

10. Centers for Disease Control and Prevention. Preliminary flu vaccine effectiveness (VE) data for 2022–2023. 2023 [cited 2024 Sep 4]. https://www.cdc.gov/flu/vaccines-work/2022-2023.html

11. Centers for Disease Control and Prevention. Weekly flu vaccination dashboard. Centers for Disease Control and Prevention. 2024 [cited 2024 Sep 4]. https://www.cdc.gov/flu/fluvaxview/dashboard/vaccination-dashboard.html

12. RSV-NET interactive dashboard. 2023 [cited 2024 Sep 4]. https://www.cdc.gov/rsv/research/rsv-net/dashboard.html

13. US Food and Drug Administration. FDA approves first respiratory syncytial virus (RSV) vaccine. 2023 [cited 2023 Aug 18]. https://www.fda.gov/news-events/press-announcements/fda-approves-first-respiratory-syncytial-virus-rsv-vaccine

14. CDC recommends a powerful new tool to protect infants from the leading cause of hospitalization. Centers for Disease Control and Prevention. 2023 [cited 2023 Aug 13]. https://www.cdc.gov/media/releases/2023/p-0803-new-tool-prevent-infant-hospitalization-.html

15. Papi A, Ison MG, Langley JM, Lee DG, Leroux-Roels I, Martinon-Torres F, et al.; AReSVi-006 Study Group. Respiratory Syncytial Virus Prefusion F Protein Vaccine in Older Adults. N Engl J Med. 2023;388:595–608. PubMed https://doi.org/10.1056/NEJMoa2209604

16. Wilkins D, Yuan Y, Chang Y, Aksyuk AA, Núñez BS, Wählby-Hamrén U, et al. Durability of neutralizing RSV antibodies following nirsevimab administration and elicitation of the natural immune response to RSV infection in infants. Nat Med. 2023;29:1172–9. PubMed https://doi.org/10.1038/s41591-023-02316-5

17. Melgar M, Britton A, Roper LE, Talbot HK, Long SS, Kotton CN, et al. Use of respiratory syncytial virus vaccines in older adults: recommendations of the Advisory Committee on Immunization Practices—United States, 2023. MMWR Morb Mortal Wkly Rep. 2023;72:793–801. PubMed https://doi.org/10.15585/mmwr.mm7229a4

18. Hammitt LL, Dagan R, Yuan Y, Baca Cots M, Bosheva M, Madhi SA, et al.; MELODY Study Group. Nirsevimab for Prevention of RSV in Healthy Late-Preterm and Term Infants. N Engl J Med. 2022;386:837–46. PubMed https://doi.org/10.1056/NEJMoa2110275

19. Nirsevimab receipt and intent for infants, United States. 2024 [cited 2024 Sep 4]. https://www.cdc.gov/vaccines/imz-managers/coverage/rsvvaxview/nirsevimab-coverage.html

20. Andrews N, Tessier E, Stowe J, Gower C, Kirsebom F, Simmons R, et al. Duration of protection against mild and severe disease by Covid-19 vaccines. N Engl J Med. 2022;386:340–50. PubMed https://doi.org/10.1056/NEJMoa2115481

21. Khoury DS, Cromer D, Reynaldi A, Schlub TE, Wheatley AK, Juno JA, et al. Neutralizing antibody levels are highly predictive of immune protection from symptomatic SARS-CoV-2 infection. Nat Med. 2021;27:1205–11. PubMed https://doi.org/10.1038/s41591-021-01377-8

22. Ferdinands JM, Rao S, Dixon BE, Mitchell PK, DeSilva MB, Irving SA, et al. Waning 2-dose and 3-dose effectiveness of mRNA vaccines against COVID-19-associated emergency department and urgent care encounters and hospitalizations among adults during periods of Delta and Omicron variant predominance—VISION Network, 10 states, August 2021–January 2022. MMWR Morb Mortal Wkly Rep. 2022;71:255–63. PubMed https://doi.org/10.15585/mmwr.mm7107e2

23. Home—COVID 19 scenario model hub [cited 2023 Aug 13]. https://covid19scenariomodelinghub.org

24. Bouchnita A, Bi K, Fox SJ, Meyers LA. Projecting Omicron scenarios in the US while tracking population-level immunity. Epidemics. 2024;46:100746. PubMed https://doi.org/10.1016/j.epidem.2024.100746

25. Respiratory Syncytial Virus (RSV) Vaccination Coverage and Intent for Vaccination. Adults 60 years and older, United States [cited 2024 Sep 4]. https://www.cdc.gov/vaccines/imz-managers/coverage/rsvvaxview/adults-60-coverage-intent.html

26. Home—Flu scenario model hub [cited 2023 Aug 13]. https://staging-extended.fluscenariomodelinghub.org

27. Fox SJ, Lachmann M, Tec M, Pasco R, Woody S, Du Z, et al. Real-time pandemic surveillance using hospital admissions and mobility data. Proc Natl Acad Sci U S A. 2022;119:119. PubMed https://doi.org/10.1073/pnas.2111870119

28. Prem K, Cook AR, Jit M. Projecting social contact matrices in 152 countries using contact surveys and demographic data. PLOS Computational Biology. 2017;13:e1005697. https://doi.org/10.1371/journal.pcbi.1005697

29. Bloom-Feshbach K, Alonso WJ, Charu V, Tamerius J, Simonsen L, Miller MA, et al. Latitudinal variations in seasonal activity of influenza and respiratory syncytial virus (RSV): a global comparative review. PLoS One. 2013;8:e54445. PubMed https://doi.org/10.1371/journal.pone.0054445

30. Cotton RD. National Oceanic and Atmospheric Administration and the environment. Arch Environ Health. 1971;22:404–5. PubMed https://doi.org/10.1080/00039896.1971.10665864

31. Mahajan S, Caraballo C, Li SX, Dong Y, Chen L, Huston SK, et al. SARS-CoV-2 infection hospitalization rate and infection fatality rate among the non-congregate population in

Connecticut. Am J Med. 2021;134:812–816.e2. PubMed https://doi.org/10.1016/j.amjmed.2021.01.020

32. Yang M-J, Rooks BJ, Le TT, Santiago IO III, Diamond J, Dorsey NL, et al. Influenza vaccination and hospitalizations among COVID-19 infected adults. J Am Board Fam Med. 2021;34(Suppl):S179–82. PubMed https://doi.org/10.3122/jabfm.2021.S1.200528

33. Update on RSV and new vaccine recommendation (2023). https://www.cdc.gov/respiratory-viruses/whats-new/rsv-update-2023-09-22.html. [2023–08–13].

34. U.S. Department of Health & Human Services. COVID-19 Reported Patient Impact and Hospital Capacity by State Timeseries (RAW) (2020). https://healthdata.gov/Hospital/COVID-19-Reported-Patient-Impact-and-Hospital-Capa/g62h-syeh/about_data. [2023–08–13].

35. Marshall NJ, Lee JL, Schroeder J, Lee WN, See J, Madjid M, et al. Influence of digital intervention messaging on influenza vaccination rates among adults with cardiovascular disease in the United States: Decentralized randomized controlled trial. J Med Internet Res. 2022;24:e38710. PubMed https://doi.org/10.2196/38710

36. Garten R, Blanton L, Elal AIA, Alabi N, Barnes J, Biggerstaff M, et al. Update: Influenza Activity in the United States During the 2017-18 Season and Composition of the 2018-19 Influenza Vaccine. MMWR Morb Mortal Wkly Rep. 2018;67:634–42. PubMed https://doi.org/10.15585/mmwr.mm6722a4

37. Abu-Raya B, Viñeta Paramo M, Reicherz F, Lavoie PM. Why has the epidemiology of RSV changed during the COVID-19 pandemic? EClinicalMedicine. 2023;61:102089. PubMed https://doi.org/10.1016/j.eclinm.2023.102089

38. Verity R, Okell LC, Dorigatti I, Winskill P, Whittaker C, Imai N, et al. Estimates of the severity of coronavirus disease 2019: a model-based analysis. Lancet Infect Dis. 2020;20:669–77. PubMed https://doi.org/10.1016/S1473-3099(20)30243-7

39. Giacchetta I, Primieri C, Cavalieri R, Domnich A, de Waure C. The burden of seasonal influenza in Italy: A systematic review of influenza-related complications, hospitalizations, and mortality. Influenza Other Respir Viruses. 2022;16:351–65. PubMed https://doi.org/10.1111/irv.12925

40. National center for health statistics mortality surveillance system [cited 2023 Aug 13]. https://gis.cdc.gov/grasp/fluview/mortality.html

41. Bonavitacola J. Study explores mortality rates and risk factors in patients hospitalized for RSV. AJMC. 2023 [cited 2023 Aug 13]. https://www.ajmc.com/view/study-explores-mortality-rates-and-risk-factors-in-patients-hospitalized-for-rsv

42. U.S. Census Bureau. Explore census data [cited 2023 Aug 13]. https://data.census.gov

43. He X, Lau EHY, Wu P, Deng X, Wang J, Hao X, et al. Temporal dynamics in viral shedding and transmissibility of COVID-19. Nat Med. 2020;26:672–5. PubMed https://doi.org/10.1038/s41591-020-0869-5

44. Macedo A, Gonçalves N, Febra C. COVID-19 fatality rates in hospitalized patients: systematic review and meta-analysis. Ann Epidemiol. 2021;57:14–21. PubMed https://doi.org/10.1016/j.annepidem.2021.02.012

45. Ma Q, Liu J, Liu Q, Kang L, Liu R, Jing W, et al. Global percentage of asymptomatic SARS-CoV-2 infections among the tested population and individuals with confirmed COVID-19 diagnosis: a systematic review and meta-analysis. JAMA Netw Open. 2021;4:e2137257. PubMed https://doi.org/10.1001/jamanetworkopen.2021.37257

46. Centers for Disease Control and Prevention. COVID-19 vaccination and case trends by age group, United States [cited 2023 Aug 13]. https://data.cdc.gov/Vaccinations/Archive-COVID-19-Vaccination-and-Case-Trends-by-Ag/gxj9-t96f/data

47. He D, Zhao S, Lin Q, Zhuang Z, Cao P, Wang MH, et al. The relative transmissibility of asymptomatic COVID-19 infections among close contacts. Int J Infect Dis. 2020;94:145–7. PubMed https://doi.org/10.1016/j.ijid.2020.04.034

48. Faes C, Abrams S, Van Beckhoven D, Meyfroidt G, Vlieghe E, Hens N; Belgian Collaborative Group on COVID-19 Hospital Surveillance. Time between symptom onset, hospitalisation and recovery or death: statistical analysis of Belgian COVID-19 patients. Int J Environ Res Public Health. 2020;17:17. PubMed https://doi.org/10.3390/ijerph17207560

49. Scobie HM, Panaggio M, Binder AM, Gallagher ME, Duck WM, Graff P, et al. Correlations and timeliness of COVID-19 surveillance data sources and indicators—United States, October 1, 2020-March 22, 2023. MMWR Morb Mortal Wkly Rep. 2023;72:529–35. PubMed https://doi.org/10.15585/mmwr.mm7219e2

50. Cohen KW, Linderman SL, Moodie Z, Czartoski J, Lai L, Mantus G, et al. Longitudinal analysis shows durable and broad immune memory after SARS-CoV-2 infection with persisting antibody

responses and memory B and T cells. Cell Rep Med. 2021;2:100354. PubMed https://doi.org/10.1016/j.xcrm.2021.100354

51. Marcotte H, Piralla A, Zuo F, Du L, Cassaniti I, Wan H, et al. Immunity to SARS-CoV-2 up to 15 months after infection. iScience. 2022;25:103743. PubMed https://doi.org/10.1016/j.isci.2022.103743

52. Kirsebom FCM, Andrews N, Stowe J, Ramsay M, Lopez Bernal J. Duration of protection of ancestral-strain monovalent vaccines and effectiveness of bivalent BA.1 boosters against COVID-19 hospitalisation in England: a test-negative case-control study. Lancet Infect Dis. 2023;23:1235–43. PubMed https://doi.org/10.1016/S1473-3099(23)00365-1

53. COVID-19 provisional counts—weekly updates by select demographic and geographic characteristics. 2023 [cited 2023 Aug 13].

54. Ghebrehewet S, MacPherson P, Ho A. Influenza. BMJ. 2016;355:i6258. PubMed https://doi.org/10.1136/bmj.i6258

55. Inpatient Hospital Stays and Emergency Department Visits Involving Influenza. 2006–2016 #253 [cited 2023 Aug 13]. https://hcup-us.ahrq.gov/reports/statbriefs/sb253-Influenza-Hospitalizations-ED-Visits-2006-2016.jsp

56. Influenza (seasonal) [cited 2023 Aug 13]. https://www.who.int/news-room/fact-sheets/detail/influenza-(seasonal)

57. Memoli MJ, Han A, Walters KA, Czajkowski L, Reed S, Athota R, et al. Influenza A reinfection in sequential human challenge: implications for protective immunity and "universal" vaccine development. Clin Infect Dis. 2020;70:748–53. PubMed https://doi.org/10.1093/cid/ciz281

58. Xie Y, Choi T, Al-Aly Z. Risk of death in patients hospitalized for COVID-19 vs seasonal influenza in Fall–Winter 2022–2023. JAMA. 2023;329:1697–9. PubMed https://doi.org/10.1001/jama.2023.5348

59. Krammer F. The human antibody response to influenza A virus infection and vaccination. Nat Rev Immunol. 2019;19:383–97. PubMed https://doi.org/10.1038/s41577-019-0143-6

60. Ferdinands JM, Fry AM, Reynolds S, Petrie J, Flannery B, Jackson ML, et al. Intraseason waning of influenza vaccine protection: evidence from the US Influenza Vaccine Effectiveness Network, 2011–12 through 2014–15. Clin Infect Dis. 2017;64:544–50. PubMed https://doi.org/10.1093/cid/ciw816

61. Osterholm MT, Kelley NS, Sommer A, Belongia EA. Efficacy and effectiveness of influenza vaccines: a systematic review and meta-analysis. Lancet Infect Dis. 2012;12:36–44. PubMed https://doi.org/10.1016/S1473-3099(11)70295-X

62. Belongia EA, Simpson MD, King JP, Sundaram ME, Kelley NS, Osterholm MT, et al. Variable influenza vaccine effectiveness by subtype: a systematic review and meta-analysis of test-negative design studies. Lancet Infect Dis. 2016;16:942–51. PubMed https://doi.org/10.1016/S1473-3099(16)00129-8

63. Centers for Disease Control and Prevention. Flu vaccine provided substantial protection this season. 2023[cited 2023 Aug 13]. https://www.cdc.gov/flu/spotlights/2022-2023/flu-vaccine-protection.htm

64. Hall CB, Douglas RG Jr, Geiman JM. Respiratory syncytial virus infections in infants: quantitation and duration of shedding. J Pediatr. 1976;89:11–5. PubMed https://doi.org/10.1016/S0022-3476(76)80918-3

65. Walsh E, Lee N, Sander I, Stolper R, Zakar J, Wyffels V, et al. RSV-associated hospitalization in adults in the USA: a retrospective chart review investigating burden, management strategies, and outcomes. Health Sci Rep. 2022;5:e556. PubMed https://doi.org/10.1002/hsr2.556

66. Sterner G. Respiratory syncytial virus in hospital cross-infection. BMJ. 1972;1:51–2. PubMed https://doi.org/10.1136/bmj.1.5791.51-d

67. Kravetz HM, Knight V, Chanock RM, Morris JA, Johnson KM, Rifkind D, et al. Respiratory syncytial virus. III. Production of illness and clinical observations in adult volunteers. JAMA. 1961;176:657–63. PubMed

68. Green M, Brayer AF, Schenkman KA, Wald ER. Duration of hospitalization in previously well infants with respiratory syncytial virus infection. Pediatr Infect Dis J. 1989;8:601–5. PubMed https://doi.org/10.1097/00006454-198909000-00007

69. Cai W, Buda S, Schuler E, Hirve S, Zhang W, Haas W. Risk factors for hospitalized respiratory syncytial virus disease and its severe outcomes. Influenza Other Respir Viruses. 2020;14:658–70. PubMed https://doi.org/10.1111/irv.12729

70. Carvajal JJ, Avellaneda AM, Salazar-Ardiles C, Maya JE, Kalergis AM, Lay MK. Host components contributing to respiratory syncytial virus pathogenesis. Front Immunol. 2019;10:2152. PubMed https://doi.org/10.3389/fimmu.2019.02152

71. Johns Hopkins Bloomberg School of Public Health. RSV, COVID-19, and flu outlook for 2023–2024. 2023 [cited 2024 Sep 4]. https://publichealth.jhu.edu/2023/rsv-covid-19-and-flu-outlook-for-2023-2024

72. Liu X, Huang J, Li C, Zhao Y, Wang D, Huang Z, et al. The role of seasonality in the spread of COVID-19 pandemic. Environ Res. 2021;195:110874. PubMed https://doi.org/10.1016/j.envres.2021.110874

73. Šmíd M, Berec L, Přibylová L, Májek O, Pavlík T, Jarkovský J, et al. Protection by vaccines and previous infection against the omicron variant of severe acute respiratory syndrome coronavirus 2. J Infect Dis. 2022;226:1385–90. PubMed https://doi.org/10.1093/infdis/jiac161

74. Abu-Raddad LJ, Chemaitelly H, Ayoub HH, AlMukdad S, Yassine HM, Al-Khatib HA, et al. Effect of mRNA vaccine boosters against SARS-CoV-2 Omicron infection in Qatar. N Engl J Med. 2022;386:1804–16. PubMed https://doi.org/10.1056/NEJMoa2200797

75. Centers for Disease Control and Prevention. COVID-19 vaccinations in the United States, jurisdiction. 2022 [cited 2023 Aug 13]. https://data. cdc. gov/Vaccinations/COVID-19-Vaccinations-in-the-United-States-Jurisdi/unsk-b7fc/data

76. Flu vaccination coverage, United States, 2022–23 influenza season. 2023 [cited 2023 Aug 13]. https://www.cdc.gov/flu/fluvaxview/coverage-2223estimates.htm

77. U.S. Food and Drug Administration Office of the Commissioner. FDA approves first respiratory syncytial virus (RSV) vaccine. 2023 [cited 2023 Aug 13]. https://www.fda.gov/news-events/press-announcements/fda-approves-first-respiratory-syncytial-virus-rsv-vaccine

78. U.S. Food and Drug Administration Office of the Commissioner. FDA approves new drug to prevent RSV in babies and toddlers 2023 [cited 2023 Aug 13]. https://www.fda.gov/news-events/press-announcements/fda-approves-new-drug-prevent-rsv-babies-and-toddlers

79. Bennett MV, McLaurin K, Ambrose C, Lee HC. Population-based trends and underlying risk factors for infant respiratory syncytial virus and bronchiolitis hospitalizations. PLoS One. 2018;13:e0205399. PubMed https://doi.org/10.1371/journal.pone.0205399

80. U.S. Department of Health and Human Services. COVID-19 reported patient impact and hospital capacity by state timeseries. 2020 [cited 2023 Aug 13]. https://catalog.data.gov/dataset/covid-19-reported-patient-impact-and-hospital-capacity-by-state-timeseries-cf58c

81. Scenario projections for SARS-CoV-2, influenza, and RSV burden in the US (2023–2024) [cited 2023 Sep 4]. https://www.researchsquare.com/article/rs-3467930/latest

82. Home—flu scenario model hub [cited 2023 Aug 13]. https://fluscenariomodelinghub.org

83. Home—scenario model hub [cited 2023 Aug 13]. https://scenariomodelinghub.org

84. Centers for Disease Control and Prevention. FluView. National, regional, and state level outpatient illness and viral surveillance [cited 2024 Sep 4]. https://gis.cdc.gov/grasp/fluview/fluportaldashboard.html

85. Influenza vaccine doses distributed, United States. 2024 [cited 2024 Sep 4]. https://www.cdc.gov/flu/fluvaxview/dashboard/vaccination-doses-distributed.html

Appendix Table 1. SARS-CoV-2 scenarios for the 2023-2024 respiratory virus season.

	No new variant	New variant (EG.5)*<br>Immune escape rate 50%
Reformulated annual vaccination recommended for all eligible groups, available beginning October 1, 2023. Age-specific vaccination rates match those from the September 2021 booster vaccination campaign.	COV-A	COV-C
Reformulated annual vaccination recommended for 65+ only, available beginning October 1, 2023. Age-specific vaccination rates match those from the September 2021 booster vaccination campaign.	COV-B	COV-D

* The relative frequency of the new variant increases exponentially and reaches 60% December 1, 2023.

Appendix Table 2. Influenza scenarios for the 2023-2024 respiratory virus season.

	H1N1 dominant<br>age-specific infection hospitalization rate based on the 2019-2020 season	H3N2 dominant<br>age-specific infection hospitalization rate based on the 2017-2018 season
Higher vaccine uptake<br>60% of US population vaccinates; VE = 40% reduction in chance of influenza-related hospitalization	INF-A	INF-C
Lower vaccine uptake<br>40% of US population vaccinates; VE = 40% reduction in chance of influenza-related hospitalization	INF-B	INF-D

Appendix Table 3. RSV scenarios for the 2023-2024 respiratory virus season.

Scenarios	Lower transmission rate<br>(pre-pandemic)	Higher transmission rate<br>(like 2022-2023)
Higher intervention<br>● Antibody immunization recommended for <8 months (100% uptake)<br>● Vaccination recommended for 60+ (56.12% uptake, based on 2022-2023 influenza vaccination)	RSV-A	RSV-C
Lower intervention<br>● No antibody immunization recommended (high risk only, 1.7% uptake)<br>● No vaccination recommended (0% coverage)	RSV-B	RSV-D

Appendix Table 4. 2023-2024 Tripledemic Projection Scenarios.

	COVID-19	Influenza	RSV
TRI-A Scenario 1: Higher medical countermeasures and lower transmissibility	COV-A	INF- A	RSV-A
TRI-B Scenario 2: Lower medical countermeasures and lower transmissibility	COV-B	INF-B	RSV-B
TRI-C Scenario 3: Higher medical countermeasures and higher transmissibility	COV-C	INF-C	RSV-C

TRI-D Scenario 4: Lower medical countermeasures and higher transmissibility	COV-D	INF-D	RSV-D

Appendix Table 5. SARS-CoV-2 model parameters.

Parameters	Value	Source
N: US population	334,994,511	ref.[42]
γ^Y: untreated symptomatic recovery rate	0.25	ref.[43]
γ^A: asymptomatic recovery rate	Equal to γ^Y	Assumption
γ_H: hospitalization recovery rate	0.1	ref.[44]
τ_I: symptomatic proportion	0.65	ref. [45]
σ: transition rate out of exposed	0.67	ref [46]
ρ^Y: pre-symptomatic to infectious transition rate	0.43	ref.[43]
ρ^A: pre-asymptomatic to infectious transition rate	Equal to ρ^Y	Assumption
θ: relative infectiousness of asymptomatic vs symptomatic infections	0.67	ref.[47]
μ_a: infection hospitalization rate (IHR), age specific	[0.0202, 0.0070, 0.0236, 0.1303, 0.2804, 0.9012]*	Initialized from ref.[31], then calibrated by fitting to ref.[34]
ζ: symptomatic to hospitalization transition rate	0.1	ref.[48]
ν_a: in-hospital mortality rate, age specific	[0.0241, 0.0133, 0.0133, 0.0133, 0.0967, 0.1631]*	Least squares fitting to ref.[40] assuming proportional to ref.[38]
π: hospitalized to death transition rate	0.2	ref.[49]
k_C: conversion rate of population immunization from natural infections	$\frac{250}{N}$	refs.[19,24,25]
k_V: conversion rate of population immunization from vaccination	$\frac{10}{N}$	refs.[19,24,25]
K_s: constant of immune saturation from natural infection	100	refs.[19,24,25]
ω_C: half life of immunity against infections following infection	8 months	ref. [50]
ω_V: half life of immunity against infections following vaccination	6 months	ref.[21]
ω_B: half life of immunity against infections following booster	3 months	ref.[22]

ω_{RB}: half life of immunity against infections following reformulated booster	Equal to ω_B	Assumption
$\omega_C^{'}$: half life of immunity against hospitalizations following infection	15 months	ref.[51]
$\omega_V^{'}$: half life of immunity against hospitalizations following vaccination	12 months	ref.[21]
$\omega_B^{'}$: half life of immunity against hospitalizations following booster	6 months	ref.[22]
$\omega_{RB}^{'}$: half life of immunity against hospitalizations following reformulated booster	Equal to $\omega_B^{'}$	Assumption
$K^I_{C,a,RB}$: reformulated booster effectiveness against infection	70%	ref.[52]
$K^H_{C,a,RB}$: reformulated booster effectiveness against hospitalizations	62%	ref.[3]
β_0: transmission rate	$N(\mu = 0.0499, \sigma = 0.0105)$	Least squares fitting to ref.[53]
τ: disease progression stochastic parameter	1	Assumption

*Age groups: [0-4, 5-11, 12-8, 19-49, 50-64, 65+]

Appendix Table 6. Influenza model parameters.

Parameters	Value	Source
N: US population	334,994,511	ref.[42]
γ: infectious recovery rate	0.25	ref.[54]
γ_H: hospitalized recovery rate	0.17	ref.[55]
σ: transition rate out exposed	0.5	ref.[56]
μ_a: infection hospitalization rate (IHR), age specific (2017-18 season)	[0.0378, 0.0091, 0.0025, 0.0038, 0.0105, 0.0680]*	Initialized from ref.[32], then calibrated by fitting to ref.[36]
μ_a: infection hospitalization rate (IHR), age specific (2019-20 season)	[0.0324, 0.0077, 0.0022, 0.0032, 0.009, 0.0583]*	Initialized from ref.[32], then calibrated by fitting to ref.[7,36]
ζ: symptomatic to hospitalization transition rate	0.25	ref.[57]
ν_a: in-hospital mortality rate, age specific	[0.0347, 0.0106, 0.0106, 0.0144, 0.0347, 0.0843]*	Least squares fitting to ref.[40] assuming proportional to ref.[39]
π: hospitalization to death transition rate	0.17	ref.[58]
ω_{inf}: half life of immunity against infections following infection	5 months	ref.[59]
ω_F: half life of immunity against infections following vaccine	3 months	ref.[57,60]

ω'_{inf}: half life of immunity against hospitalizations following infection	5 months	ref.[59]
ω'_{F}: half life of immunity against hospitalizations following vaccine	3 months	ref.[57,60]
$K^{I}_{F,a,V}$: vaccine effectiveness against infection, age specific	[52%, 52%,52%, 36%, 36%, 24%]*	ref.[61,62]
$K^{H}_{F,a,V}$: vaccine effectiveness against hospitalization, age specific	[68%, 68%, 68%, 51%, 51%, 35%]*	ref.[63]
β_0: transmission rate	$N(\mu$ = 0.0493, σ = 0.0112)	Least squares fitting to ref.[34]

*Age groups: [0-4, 5-11, 12-8, 19-49, 50-64, 65+]

Appendix Table 7. Parameter values for RSV model

Parameters	Value	Source
N: US population	334,994,511	ref.[42]
γ: infectious recovery rate	0.15	ref.[64]
γ_H: hospitalized recovery rate	0.17	ref.[65]
σ: transition rate out of exposed	0.2	ref.[66,67]
μ_a: infection hospitalization rate (IHR), age specific	[0.0215, 0.0014, 0.0002, 0.0002, 0.0001, 0.0003, 0.0012]*	Initialized from ref[33], calibrated by fitting to ref.[12]
ζ: symptomatic to hospitalization transition rate	0.147	ref.[68]
ν_a: in-hospital mortality rate, age specific	[0.0411, 0.0321, 0.0121, 0.0121, 0.0170, 0.0411, 0.1002]*	ref.[41]
π: hospitalization to death transition rate	0.17	ref.[69]
ω_ι: half life of immunity against infections following infection	Within first 6 months: 8.3 months<br>After 6 months: 4 months	ref.[70]
ω_R: half life of immunity against infections following monoclonal antibodies for infants	2 months	ref.[18]
ω_R: half life of immunity against infections following vaccination for older population	7 months	ref.[15]
ω'_ι: half life of immunity against hospitalizations following infection	Within first 6 months: 8.3 months<br>After 6 months: 4 months	ref.[70]
ω'_R: half life of immunity against hospitalizations following monoclonal antibodies for infants	2 months	ref.[18]
ω'_R: half life of immunity against hospitalizations following vaccination for older population	7 months	ref.[15]
$K^{I}_{R,a,V}$: RSV monoclonal antibody effectiveness against infection	Considered an uniform random value between 0% and 74.5% for children under 8 months	ref.[15, 18]
$K^{H}_{R,a,V}$: RSV monoclonal antibody effectiveness against hospitalization	62.1% for children under 8 months	ref.[15, 18]

$K^{I}_{R,a,V}$: RSV vaccine effectiveness against infection	Considered an uniform random value between 0% and 71.7% for adults over 60	ref.[15, 18]
$K^{H}_{R,a,V}$: RSV vaccine effectiveness against hospitalization	94.1% for adults over 60	ref.[15, 18]
β_0: transmission rate	$N(\mu = 0.08, \sigma = 0.005)$	Least squares fitting to ref[12]

*Age groups: [0-1, 2-4, 5-11, 12-18, 19-49, 50-64, 65+]

Appendix Table 8. Validation of the COVID-19, Influenza, and RSV models. Each model was validated via retrospective comparison between projected versus reported hospital admissions in the US. In most cases, the scenario assumed by the model was developed prior to viewing data from the projection period. Values in brackets are 95% projection intervals.

	Date range		Date of peak hospitalizations		Magnitude of peak		Total hospitalizations	
MODEL	Training	Testing	Actual	Model	Actual	Model	Actual	Model
COVID-19 BA.4/ BA.5 in US	Feb 28 - Jul 5, 2022	Jul 5 - Oct 31, 2022	July 25	Aug 6 [Aug 1 - Aug 13]	6,177	5,696 [4,835 - 7,135]	546,129	582,718 [420,900 - 798,172]
Flu Scenario Modeling Hub Round 3	Aug 1 - Dec 3, 2022	Dec 4, 2022 - June 3, 2023	Nov 29	Dec 1 [Nov 28 - Dec 3]	3,423	3,616 [3,369 - 3,900]	125,868	140,419 [111,378 - 251, 655]
RSV	Oct 1, 2018 - May 5, 2019; May 6 - Jul 15, 2023	Jul 16 2019 - Apr 23, 2020	Jan 4	Dec 18 [Dec 8 - Jan 20]	1,294	1,145 [290 - 1,573]	102,353	78,800 [24,376 - 177,402]

Appendix Table 9. Projected age-specific cumulative COVID-19 hospitalizations in the US between 09/01/2023 and 03/30/2024 under the four scenarios described in Table A.1. Values in each cell are medians and 95% prediction intervals based on 200 stochastic simulations.

	Age group						Total
	0-4	5-11	12-18	19-49	50-64	65+	
COV-A	10,224 [8,165-12,003]	4,470 [3,465-5,387]	9,032 [7,218-10,588]	97,301 [78,990-112,808]	152,910 [124,101-177,082]	323,950 [261,943-375,738]	598,153 [485,608-692,097]
COV-B	10,818 [8,682-12,691]	4,663 [3,631-5,617]	9,519 [7,652-11,160]	137,570 [112,419-159,344]	213,628 [174,214-246,864]	347,961 [281,994-402,956]	724,098 [590,601-837,091]
COV-C	18,879 [15,581-21,664]	7,984 [6,478-9,324]	12,606 [10,342-14,519]	171,692 [142,148-196,287]	257,938 [213,818-294,864]	547,404 [449,379-627,594]	1,016,745 [839,736-1,162,735]
COV-D	20,352 [16,870-23,266]	8,446 [6,888-9,813]	13,482 [11,132-15,512]	270,856 [225,946-308,387]	398,726 [333,448-454,017]	598,077 [493,311-683,472]	1,310,106 [1,089,610-1,492,837]

Appendix Table 10. Projected age-specific cumulative influenza hospitalizations in the US between 09/01/2023 and 04/23/2024 under the four scenarios described in Table A.2. Values in each cell are medians and 95% prediction intervals based on 200 stochastic simulations.

	Age group						Total
	0-4	5-11	12-18	19-49	50-64	65+	
INF-A	6,094 [312-15,541]	5,225 [236-13,291]	1,065 [25-3,051]	43,519 [2,753-104,598]	36,526 [2,236-88,239]	88,736 [5,269-215,949]	182,546 [10,970-438,289]
INF-B	6,795 [483-16,592]	5,836 [390-14,091]	1,180 [45-3,188]	51,308 [4,258-115,780]	42,241 [3,478-96,259]	101,100 [8,112-230,975]	209,765 [17,146-475,218]

	Age group						Total
	0-4	5-11	12-18	19-49	50-64	65+	
INF-C	2,725 [126-7,112]	4,092 [198-10,394]	1,825 [69-4,778]	40,296 [2,843-93,711]	43,379 [3,309-111,283]	258,451 [18,303-606,537]	355,622 [25,013-832,407]
INF-D	3,070 [226-7,375]	4,691 [353-10,830]	1,997 [114-4,856]	47,466 [4,541-101,502]	55,301 [5,191-118,832]	294,471 [27,083-632,765]	407,559 [37,818-874,205]

Appendix Table 11. Projected age-specific cumulative RSV hospitalizations in the US between 09/01/2023 and 04/23/2024 under the four scenarios described in Table A.3. Values in each cell are medians and 95% prediction intervals based on 200 stochastic simulations.

	Age group							Total
	0-1	2-4	5-11	12-18	19-49	50-64	65+	
RSV-A	49,849 [27,811-82,974]	6,489 [3,229-11,480]	2,316 [950-4,172]	209 [17-595]	2,655 [1,234-4,715]	4,702 [2,362-8,134]	7,936 [4,139-13,564]	74,525 [42,078-121,772]
RSV-B	60,978 [33,201-101,866]	6,584 [3,385-11,612]	2,326 [952-4,112]	210 [15-609]	2,674 [1,226-4,751]	5,584 [2,856-9,436]	16,382 [8,636-27,511]	95,044 [52,258-157,044]
RSV-C	87,858 [58,996-124,465]	9,870 [5,987-14,759]	3,125 [1,636-4,877]	320 [49-763]	3,627 [2,054-5,678]	6,660 [4,161-9,804]	12,847 [8,142-18,411]	124,403 [85,069-174,587]
RSV-D	95,518 [64,128-135,993]	9,961 [6,044-14,929]	3,142 [1,617-4,849]	320 [52-770]	3,644 [2,049-5,693]	7,665 [4,833-11,208]	14,973 [23,770-34,032]	144,121 [97,353-203,466]

Appendix Table 12. Reported age-specific COVID-19[80], influenza[80], and RSV[12] hospitalizations in the US between 09/01/2022 and 03/30/2023. The first row (TRI) provides the sum across all three viruses. The RSV hospitalizations data are collected from the RSV-NET, which are scaled up from a network of sites in acute-care hospitals across 58 counties in 12 states during the October 1–April 30 season each year[12].

	Age group						Total
	0-4	5-11	12-18	19-49	50-64	65+	
TRI 2022-2023	149,663	19,131	12,548	176,345	207,416	647,207	1,212,312
COVID 2022-2023	18,695	5,660	7,491	132,527	151,061	513,009	828,443
Flu 2022-2023	20,974	5,106	2,918	36,229	44,957	98,729	208,915
RSV 2022-2023	109,994	8,365	2,139	7,589	11,398	35,469	174,954

Appendix Table 13. Reported age-specific COVID-19[80], influenza[80], and RSV[12] hospitalizations in the US between 09/01/2023 and 03/30/2024. The first row (TRI) provides the sum across all three viruses. The RSV hospitalizations data are collected from the RSV-NET, which are scaled up from a network of sites in acute-care hospitals across 58 counties in 12 states during the October 1–April 30 season each year[12].

	Age group						Total
	0-4	5-11	12-18	19-49	50-64	65+	
TRI 2023-2024	117,875	22,103	9,851	133,460	172,503	551,018	1,006,810
COVID 2023-2024	16,449	4,350	4,935	80,504	103,620	402,758	612,616
Flu 2023-2024	16,331	12,305	2,901	42,145	50,207	91,778	215,667
RSV 2023-2024	85,095	5,448	2,015	10,811	18,676	56,482	178,527

Appendix Table 14. Projected date and value of maximum daily COVID-19 hospital admissions in the US between 09/01/2023 and 03/30/2024 under the four scenarios described in Table A.1. Dates in each cell are medians and 95% prediction intervals based on 200 stochastic simulations.

	Date of peak	Value of peak
COV-A	10/28/2023 [10/20/2023, 11/02/2023]	4,668 [3,825, 5,346]

COV-B	11/02/2023 [10/27/2023, 11/17/2023]	5,589 [4,623, 6,396]
COV-C	10/22/2023 [10/21/2023, 11/02/2023]	8,158 [6,831, 9,162]
COV-D	10/25/2023 [10/21/2023, 11/02/2023]	9,759 [8,286, 10,931]
Reported	12/29/2023	5,341

Appendix Table 15. Projected date of maximum daily influenza hospital admissions in the US between 09/01/2023 and 03/30/2024 under the four scenarios described in Table A.2. Dates in each cell are medians and 95% prediction intervals based on 200 stochastic simulations.

	Date of peak	Value of peak
INF-A	12/25/2023 [11/29/2023, 01/18/2024]	2,678 [247, 7,665]
INF-B	12/14/2023 [11/21/2023, 01/25/2024]	4,128 [393, 8,702]
INF-C	12/16/2023 [11/22/2023, 01/28/2024]	6,928 [572, 14,978]
INF-D	12/10/2023 [11/16/2023, 01/12/2024]	8,934 [911, 16,715]
Reported	12/28/2023	3,137

Appendix Table 16. Projected date of maximum daily RSV hospital admissions in the US between 09/01/2023 and 03/30/2024 under the four scenarios described in Table A.3. Dates in each cell are medians and 95% prediction intervals based on 200 stochastic simulations.

	Date of peak	Value of peak
RSV-A	12/15/2023 [12/07/2023, 01/03/2024]	961 [519, 1,461]
RSV-B	12/12/2023 [12/05/2023, 12/25/2023]	1,466 [790, 2,058]
RSV-C	11/21/23 [11/18/23, 11/29/23]	1,733 [1,318, 2,171]
RSV-D	11/16/23 [11/14/23, 11/29/23]	2,384 [1,777, 2,902]
Reported	12/30/2023	1,911

Appendix Table 17. Age-specific cumulative COVID-19, influenza, and RSV hospitalizations in the US during the 2022-2023 tripledemic (from 09/01/2022 to 03/30/2023) and projected for the 2023-2024 respiratory virus season (from 09/01/2023 to 03/30/2024) under four plausible scenarios.

	Age group						Total
	0-4	5-11	12-18	19-49	50-64	65+	
2022-23*	149,663	19,131	12,548	176,345	207,416	647,207	1,212,312
2023-24#	117,875	22,103	9,851	133,460	172,503	551,018	1,066,810
TRI-A	72,656 [42,777-117,008]	12,011 [4,651-22,849]	10,306 [7,260-14,234]	143,475 [82,976-222,121]	194,138 [128,699-273,455]	420,622 [271,350-605,251]	855,224 [538,656-1,252,158]
TRI-B	85,174 [48,950-137,752]	12,825 [4,972-23,820]	10,909 [7,712-14,957]	191,552 [117,903-279,874]	261,453 [180,547-352,559]	465,443 [298,742-661,442]	1,028,907 [660,005-1,469,353]
TRI-C	119,332 [84,573-163,111]	15,200 [8,312-24,595]	14,751 [10,460-20,059]	215,615 [147,044-295,676]	307,977 [221,287-415,950]	818,702 [475,824-1,252,541]	1,496,769 [949,818-2,169,729]
TRI-D	128,901 [91,185-176,595]	16,278 [8,857-25,491]	15,798 [11,298-21,138]	321,966 [232,536-415,582]	461,692 [343,472-584,057]	916,318 [535,366-1,350,269]	1,861,786 [1,224,781-2,570,507]

*Reported age-specific cumulative COVID-19[80], Influenza[80], and RSV[12] hospitalizations in the US between 09/01/2022 and 03/30/2023.

#Reported age-specific cumulative COVID-19[80], Influenza[80], and RSV[12] hospitalizations in the US between 09/01/2023 and 03/30/2024.

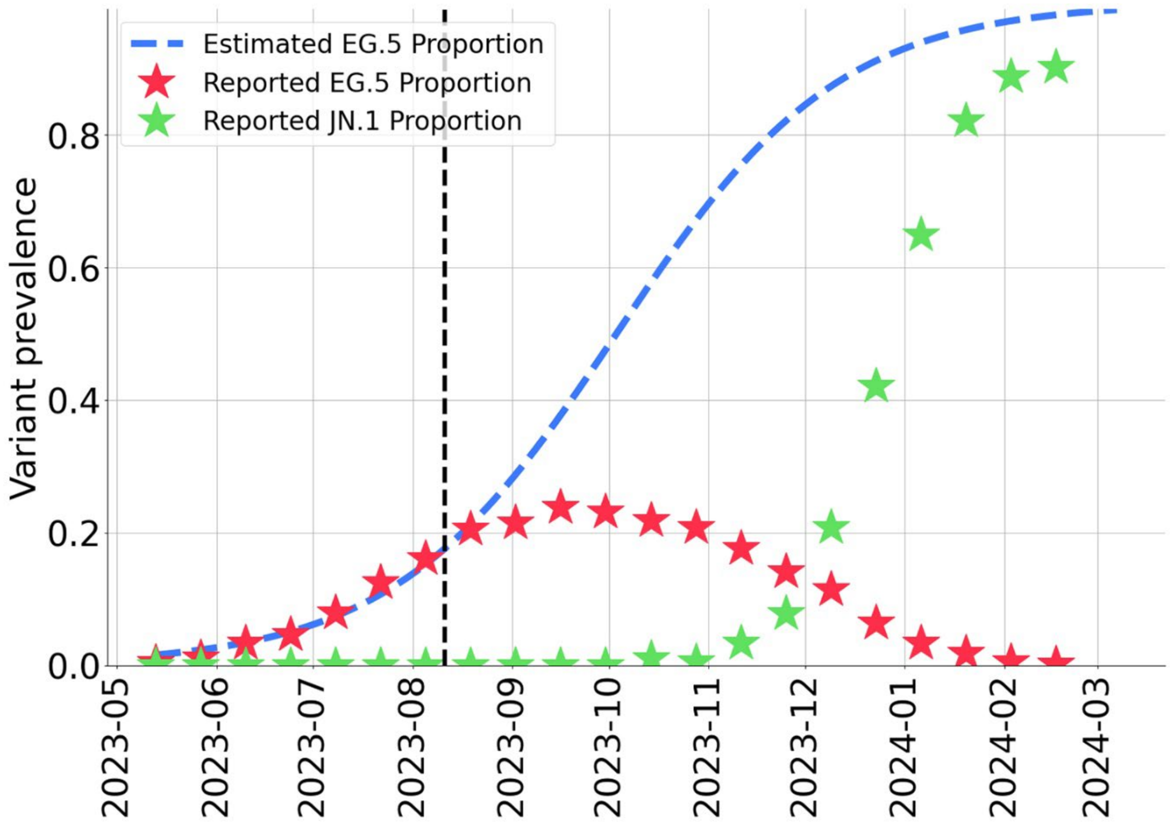

Appendix Figure 1. Estimated ascent of the SARS-CoV-2 Omicron subvariant EG.5 and JN.1 in the US. Values represent the proportion of cases caused by the Omicron subvariant EG.5. Stars indicate the reported proportion of EG.5 (red) and JN.1 (green) in a sample of US COVID-19 specimens[4]. The blue dashed line shows the fitted logistic curve used to model COVID-19 scenarios with the EG.5 variant for the projections. The model was fit to data prior to the date indicated by the vertical black dashed line.

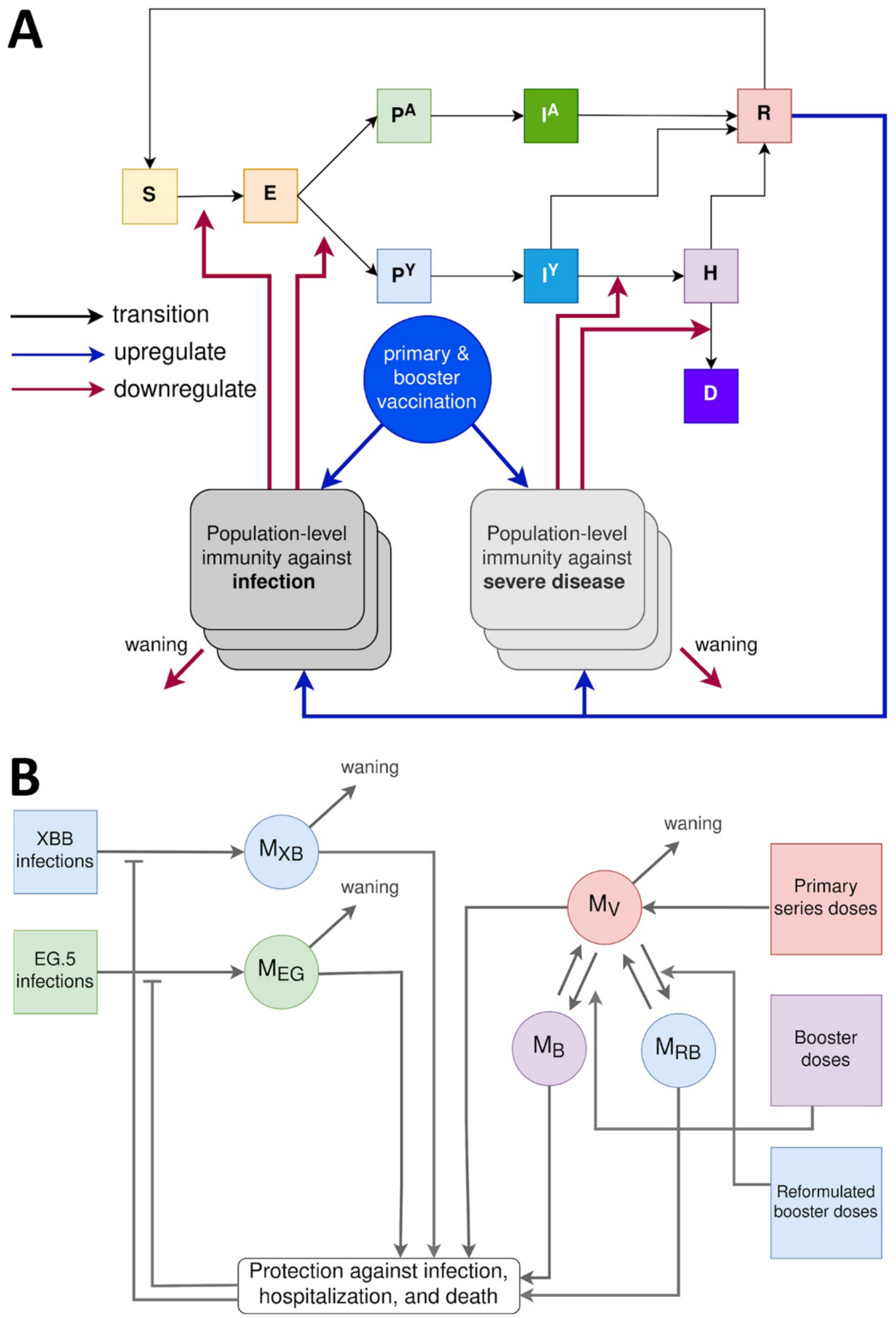

Appendix Figure 2. Schematic representation of SARS-CoV-2 transmission model that tracks population-level immunity against multiple variants derived from natural infection and vaccination for six age groups. (A) Susceptible individuals (S) move to the exposed state (E) when they get infected. Exposed individuals transition into either the pre-symptomatic (P^Y) or the pre-asymptomatic (P^A) compartment. Pre-asymptomatic cases first transition to the infectious asymptomatic compartment (I^A) and then to the recovered compartment (R) where they are fully immune to reinfection until they transition

back to being susceptible. Pre-symptomatic individuals first move to the symptomatic compartment (I^Y); a fraction of those individuals move directly to the recovered compartment, while the remaining transition to the hospitalized compartment (H). Hospitalized cases either recover (R) or die (D). Recovered individuals enjoy a short period of full immunity before returning to the susceptible compartment (S). For each type of immune exposure (i.e., infection with a specific variant or receipt of a specific type of vaccine dose), the model uses two state variables to track the resulting population-level average protection against infection and severe disease. These variables increase as individuals recover from infections and receive vaccines and they decrease according to waning (half-life) parameters, specific to each exposure type. Immunity state variables modify overall rates of infection and risk of hospitalization/death with efficacies that can vary depending on currently circulating virus variants and the age group of the exposed individual. Variables tracking population-level immunity can be readily modified to capture immunity with respect to future variants as well as multiple types of vaccines and boosters. **(B)** Infection upregulates population immunity depending on the relative frequency of each variant among active infections. These are given by M_{XB} and M_{EG}. The administration of a primary vaccine doses, booster doses and reformulated booster increases the vaccination-derived immunity and these are represented as M_V, M_B, M_{RB}. We model a separate set of infection and immunity variables for each of the following age groups: 0-4, 5-11, 12-18, 19-49, 50-64, and 65+.

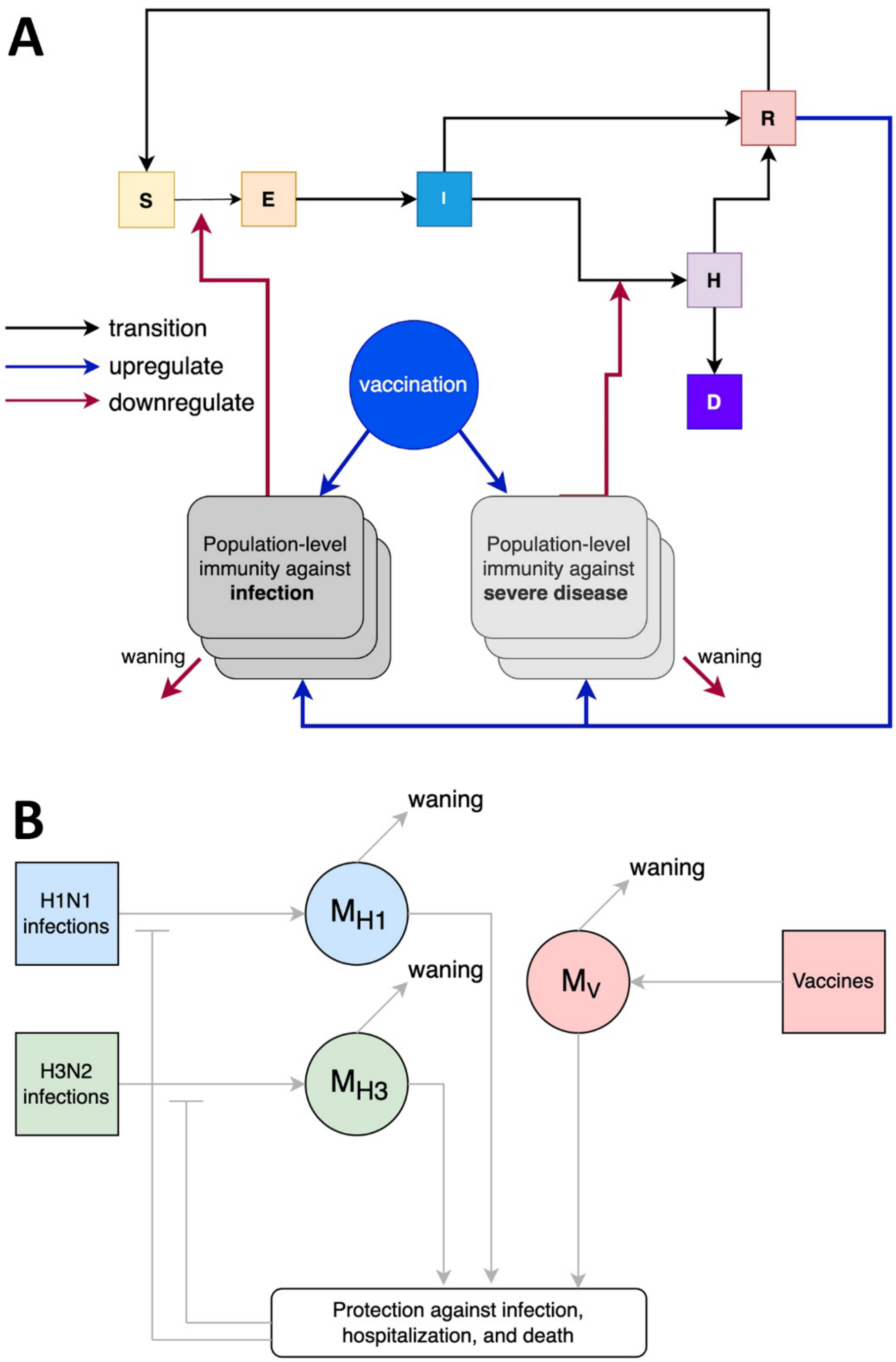

Appendix Figure 3. Schematic representation of influenza transmission model that tracks population-level immunity against two influenza subtypes derived from natural infection and vaccination for six age groups. (A) Susceptible individuals (S) move to the exposed state (E) upon infection. Exposed individuals transition to the infectious compartment (I); a fraction of individuals move directly from (I) to the recovered compartment (R), while the remaining transition to the hospitalized compartment (H). Hospitalized cases either recover (R) or die (D). Recovered individuals eventually

become partially susceptible again and move into the susceptible compartment (S). For each type of immune exposure (i.e., infection with a specific subtype or receipt of vaccine), the model uses two state variables to track the resulting population-level average protection against infection and severe disease. These variables increase as individuals recover from infections and receive vaccines and they decrease according to waning (half-life) parameters, specific to each exposure type. Immunity state variables modify overall rates of infection and risk of hospitalization/death with efficacies that can vary depending on currently circulating virus subtypes and the age group of the exposed individual. **(B)** Infection upregulates population immunity depending on the relative frequency of each subtype among active infections. These are given by M_{H1} and M_{H3}. The administration of vaccine increases vaccine-derived immunity, represented as M_V. We model a separate set of infection and immunity variables for each of the following age groups: 0-4, 5-11, 12-18, 19-49, 50-64, and 65+.

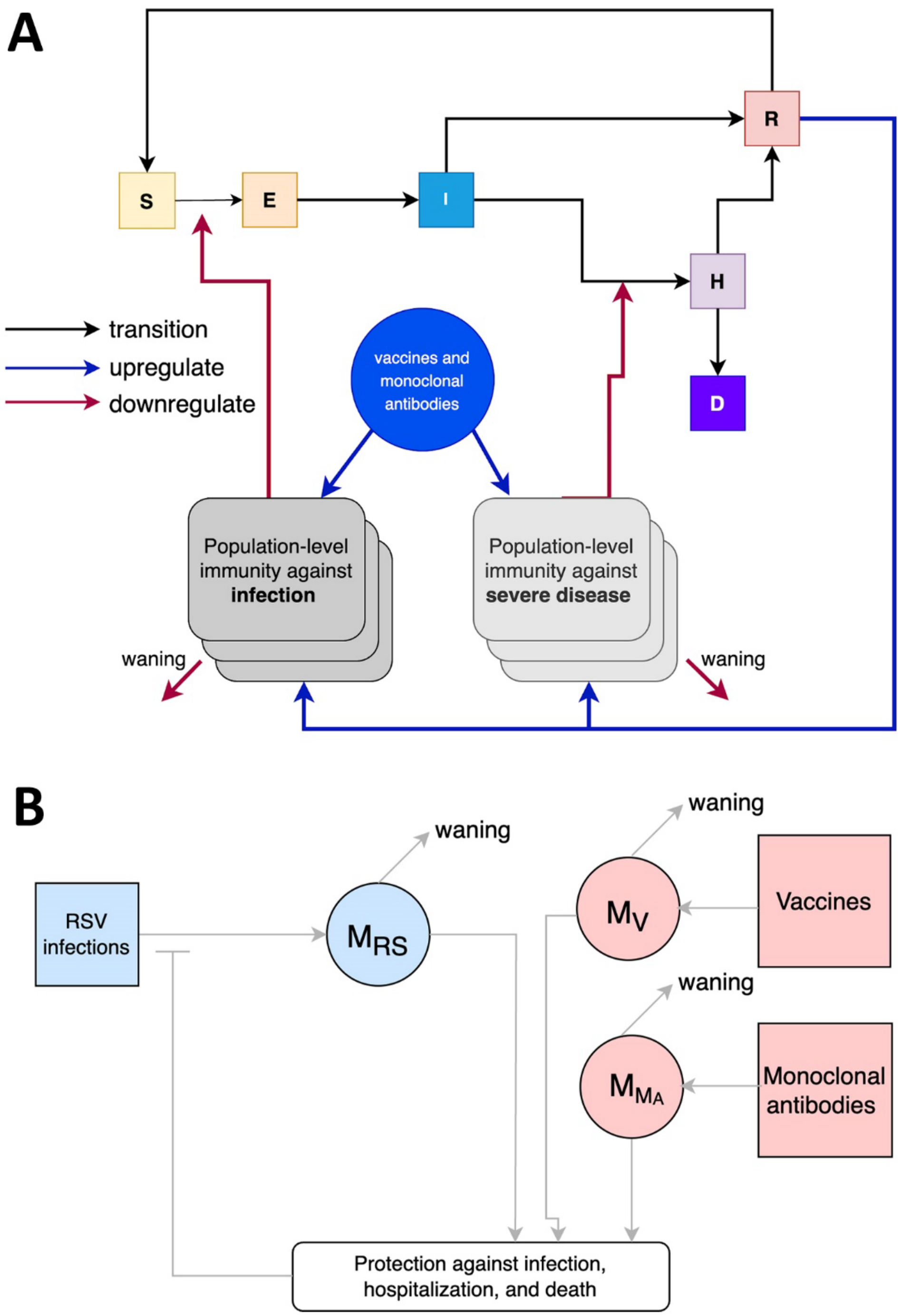

Appendix Figure 4. Schematic representation of RSV transmission model that tracks population-level immunity derived from natural infection, vaccination, and monoclonal antibodies across seven age groups. (A) Susceptible individuals (S) move to the exposed state (E) when they get infected. Exposed individuals transition to the infectious compartment (I); a fraction of individuals move directly from (I) to the recovered compartment (R), while the remaining transition to the hospitalized compartment (H). Hospitalized cases either recover (R) or die (D). Recovered individuals eventually become partially

susceptible again and move into the susceptible compartment (S). For each type of immune exposure (i.e., infection with RSV or receipt of vaccine or monoclonal antibodies), the model uses two state variables to track the resulting population-level average protection against infection and severe disease. These variables increase as individuals recover from infections or receive vaccines and monoclonal antibodies and then decrease according to waning (half-life) parameters specific to each exposure type. Immunity state variables modify overall rates of infection and risk of hospitalization/death with efficacies that can vary depending on the circulating virus and the age group of the exposed individual. **(B)** Infection upregulates population immunity depending on RSV infections depicted by M_{RS}. The administration of vaccines and monoclonal antibodies increases the vaccine-derived immunity represented as M_V and M_{M_A} . We model a separate set of infection and immunity variables for each of the following age groups: 0-1, 2-4, 5-11, 12-18, 19-49, 50-64, and 65+.

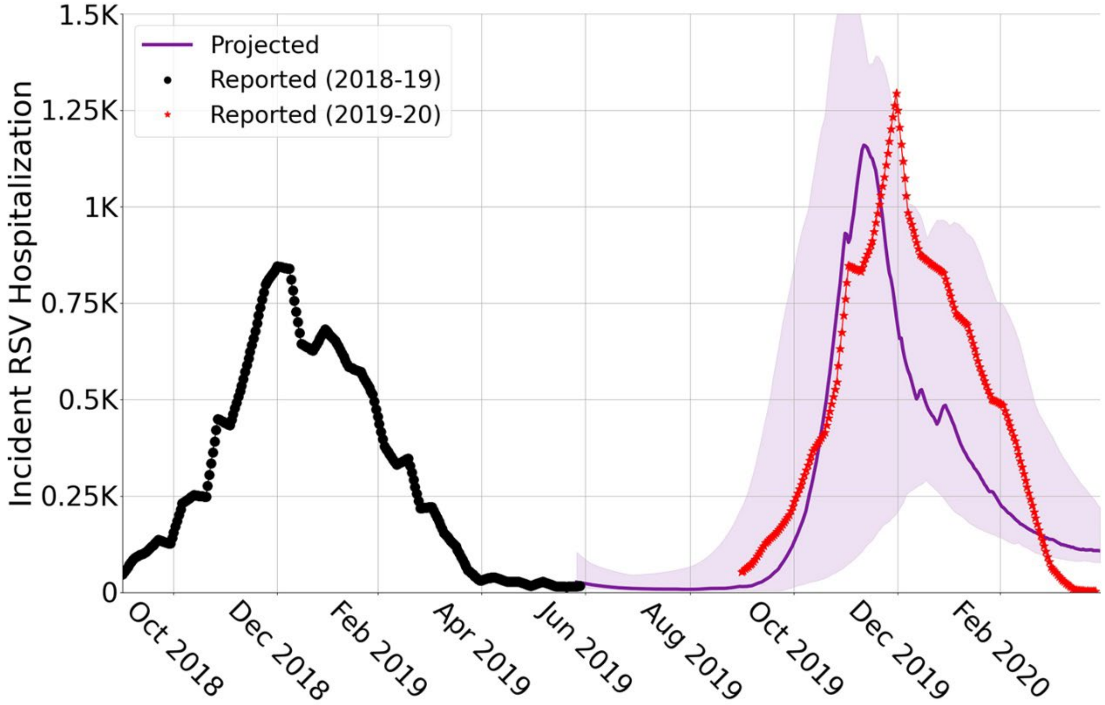

Appendix Figure 5. Absolute humidity data of the United States stratified to the day of a year.

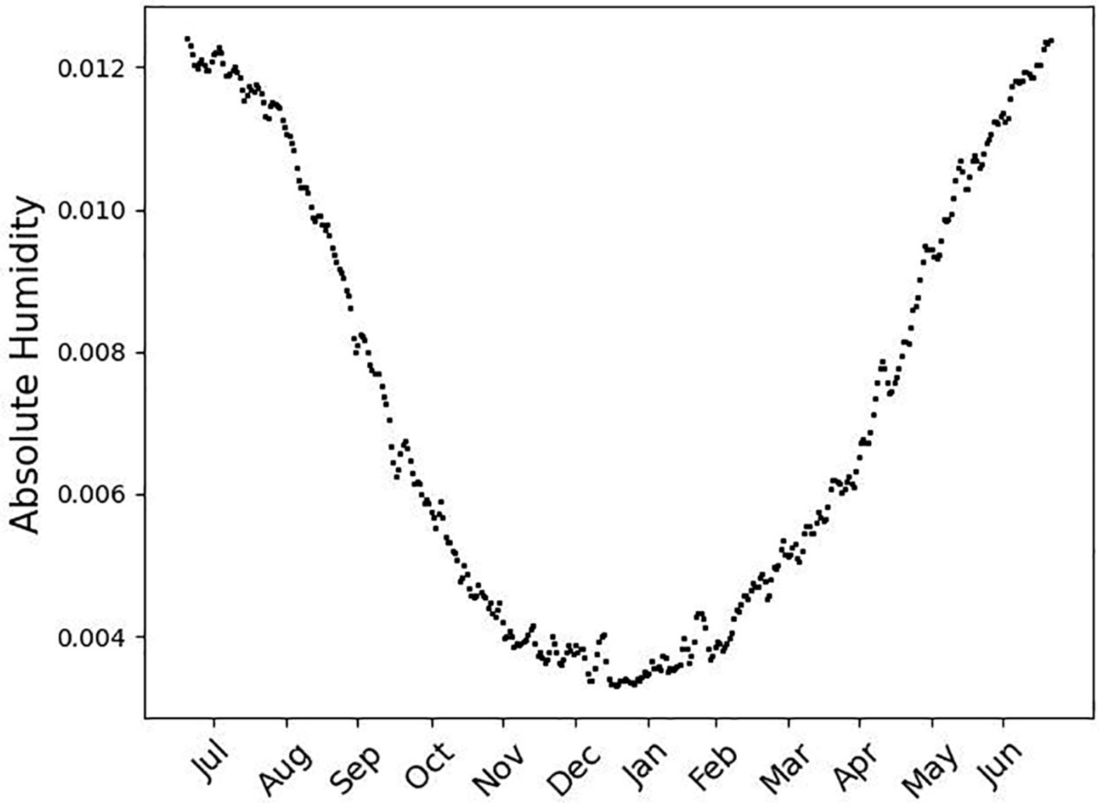

Appendix Figure 6. Comparison between retrospective RSV model projections and observed RSV hospitalization data for the US. The model was first fit to RSV hospitalization data from the 2018-2019 season[12] to estimate the level of infection-acquired immunity and transmission rate at the end of the season. Additionally, data from the 2022-23 season between May 6 2023 and July 15 2023 was used and appended with the hospitalization data from October 1, 2018 and May 5, 2019 to obtain the entire fitting period to bridge the gap in missing data between May 6 2019 and October 1 2029. The hospitalizations were then projected from July 16, 2019 through April 23, 2020. We then compared these projected results between October 1, 2019 and April 23, 2020 with the reported 2019-2020 hospitalizations. Values are a seven-day average number of hospital admissions attributable to RSV infections. The solid lines and shaded ribbons represent median and 95% prediction intervals across 200 stochastic simulations, respectively. Black dots and red dots are reported seven-day average hospital admissions for RSV in 2018-2019 and 2019-2020 seasons respectively.

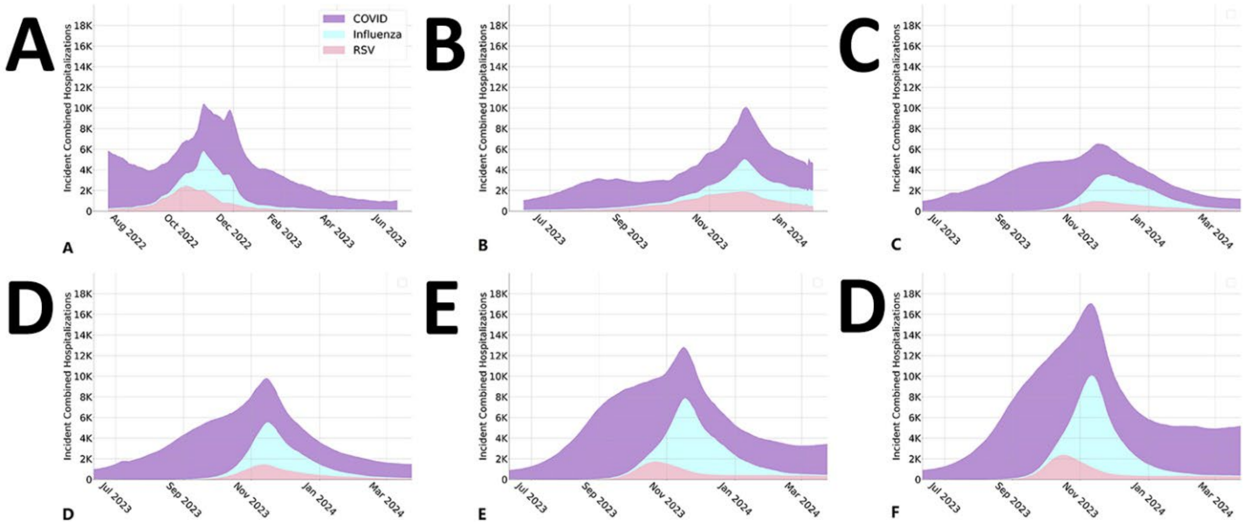

Appendix Figure 7. Reported and projected COVID-19, influenza, and RSV seven-day average hospital admissions in the US. Daily hospital admissions (A) during the 2022-2023 respiratory virus season (from August 8, 2022 to July 10, 2023)[34, 12], and (B) during the 2023-2024 respiratory virus season (from July 11, 2023 to February 17, 2024)[34, 12]. The other four panels provide projected hospitalizations from July 11, 2023 to April 23, 2024 under the four tripledemic scenarios described in Table A.4, from optimistic (lower transmission rates paired with higher impact of immunization interventions) to pessimistic (higher transmission rates paired with lower impact of immunization interventions): (C) TRI-A, (D) TRI-B, (E) TRI-C, and (F) TRI-D. Purple, blue and pink shading correspond to SARS-CoV-2, influenza, and RSV, respectively. Values are median estimates across 200 stochastic simulations.

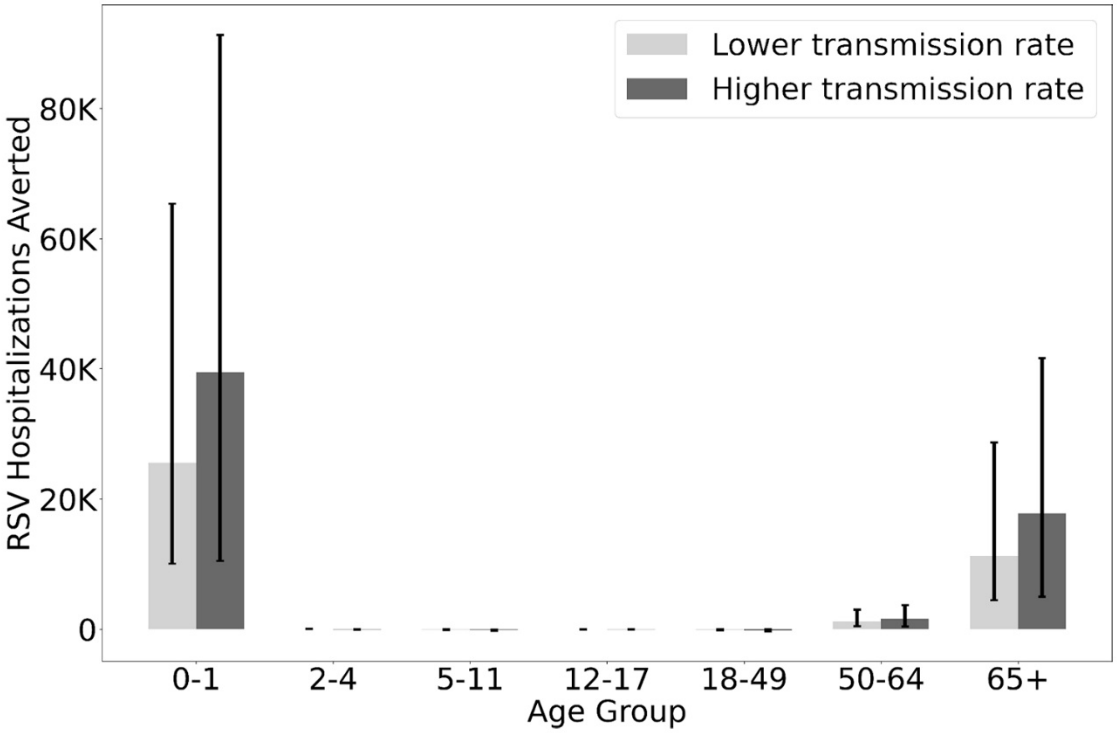

Appendix Figure 8. Projected age-specific RSV hospitalizations averted by new RSV vaccines and monoclonal antibodies between September 1, 2023 and April 23, 2024 in the US assuming a pre-pandemic transmission rate (light gray) or a higher transmission rate similar to the 2022-2023 RSV season (dark gray). Bar heights and error lines correspond to median and 95% prediction interval, across 200 pairs of stochastic simulations, in which one simulation assumes that adults over age 60 receive the RSV vaccine at rates based on historical influenza vaccination patterns and nearly all infants under age eight months receive the new monoclonal antibodies and the other simulation assumes that nobody receives the RSV vaccine and only high risk infants receive the monoclonal antibodies.

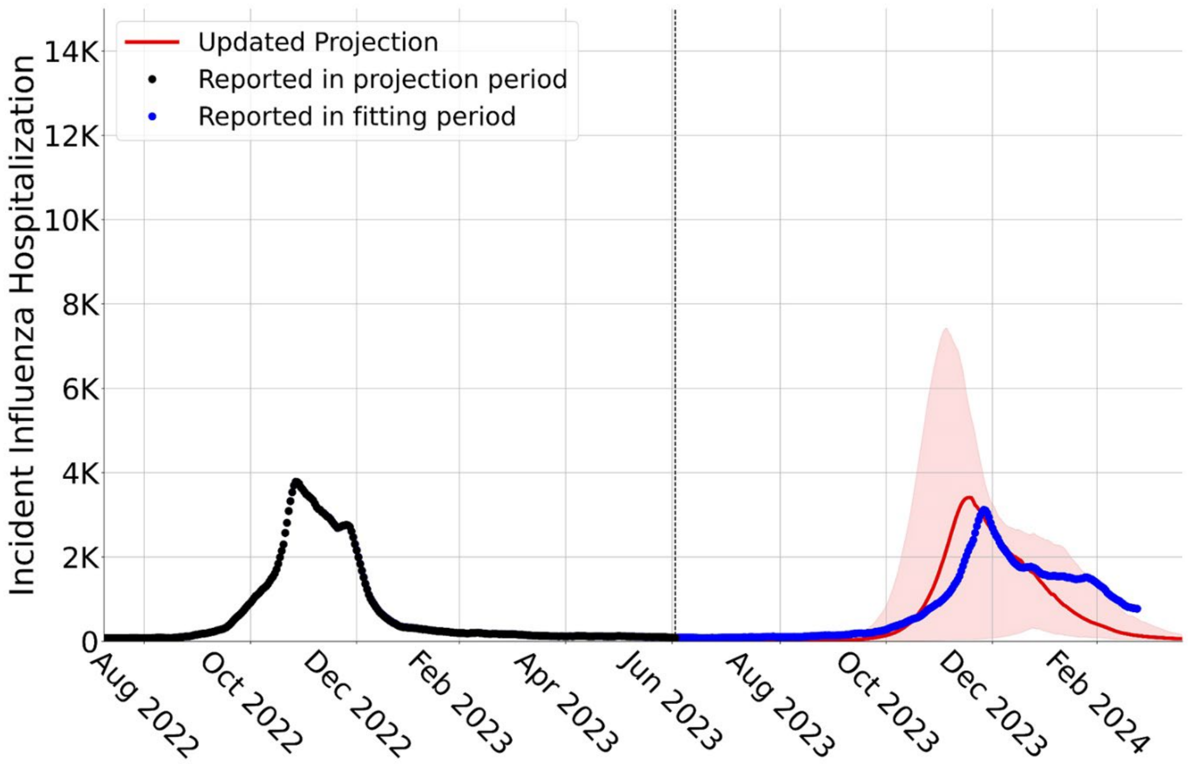

Appendix Figure 9. Post hoc projected and observed daily hospital admissions in the US attributable to influenza infections from September 1, 2023 to April 23, 2024. Projections are based on a scenario retrospectively constructed to match the dominant subtype and vaccination rates from the 2023-2024 influenza season. Values are a seven-day average number of hospital admissions attributable to influenza infections. The solid lines and shaded ribbons represent median and 95% prediction intervals across 200 stochastic simulations, respectively. Black and blue dots indicate the reported seven-day average hospital admissions for influenza prior to and during the projection period[12,34], respectively.

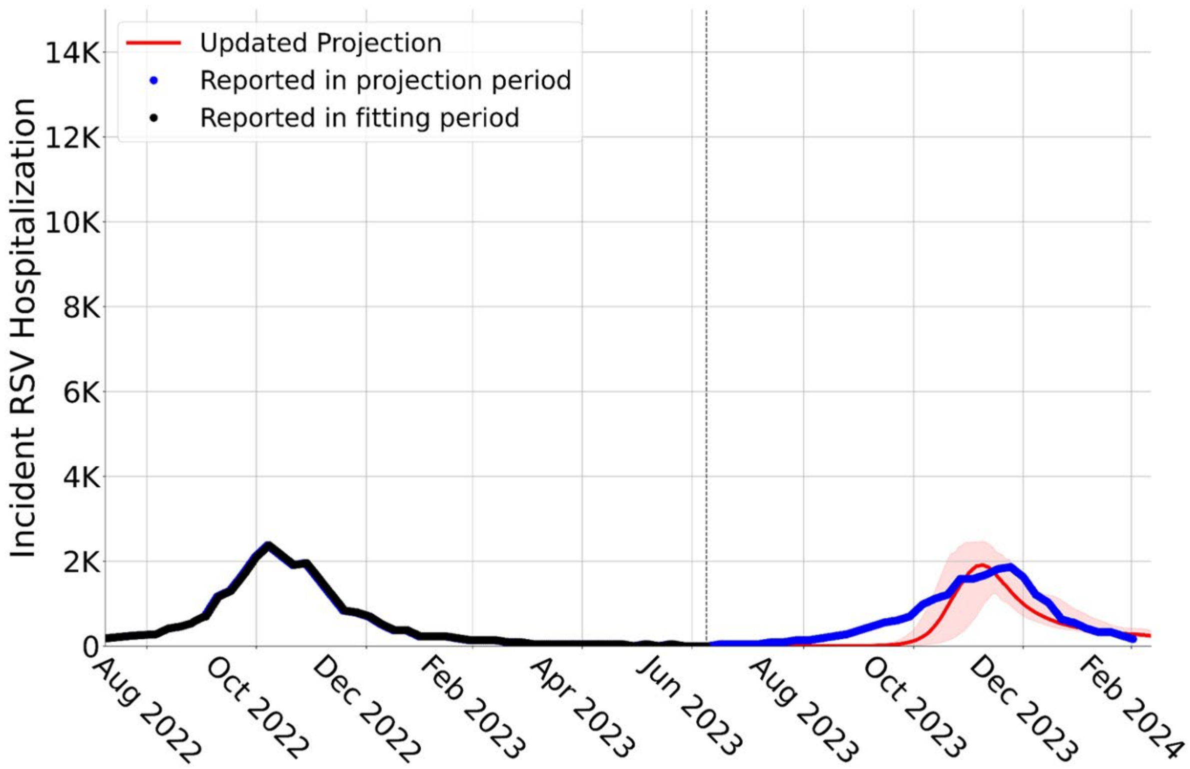

Appendix Figure 10. Post hoc projected and observed daily hospital admissions in the US attributable to RSV infections from September 1, 2023 to April 23, 2024. Projections are based on a scenario retrospectively constructed to match the observed RSV immunization rates and estimated higher rate of adult-to-adult transmission during the 2023-2024 influenza season. Values are a seven-day average number of hospital admissions attributable to RSV infections. The solid lines and shaded ribbons represent median and 95% prediction intervals across 200 stochastic simulations, respectively. Black and blue dots indicate the reported seven-day average hospital admissions for RSV prior to and during the projection period[12,34], respectively.
